# A patient-based medaka *alg2* mutant as a model for hypo-*N*-glycosylation

**DOI:** 10.1242/dev.199385

**Published:** 2021-06-07

**Authors:** Sevinç Gücüm, Roman Sakson, Marcus Hoffmann, Valerian Grote, Clara Becker, Kaisa Pakari, Lars Beedgen, Christian Thiel, Erdmann Rapp, Thomas Ruppert, Thomas Thumberger, Joachim Wittbrodt

**Affiliations:** 1 COS, Centre for Organismal Studies Heidelberg, Heidelberg University, 69120 Heidelberg, Germany; 2 HBIGS, Heidelberg Biosciences International Graduate School, Heidelberg University, 69120 Heidelberg, Germany; 3 Core facility for Mass Spectrometry and Proteomics, Zentrum für Molekulare Biologie der Universität Heidelberg (ZMBH), DKFZ-ZMBH Alliance, 69120 Heidelberg, Germany; 4 Max Planck Institute for Dynamics of Complex Technical Systems, 39106 Magdeburg, Germany; 5 Center for Child and Adolescent Medicine, Department Pediatrics I, Heidelberg University, 69120 Heidelberg, Germany; 6 glyXera GmbH, 39120 Magdeburg, Germany

**Keywords:** CDG, Human hypomorphic mutations, Disease model, Glycosylation, Medaka, Retinitis pigmentosa

## Abstract

Defects in the evolutionarily conserved protein-glycosylation machinery during embryonic development are often fatal. Consequently, congenital disorders of glycosylation (CDG) in human are rare. We modelled a putative hypomorphic mutation described in an alpha-1,3/1,6-mannosyltransferase (ALG2) index patient (ALG2-CDG) to address the developmental consequences in the teleost medaka (*Oryzias latipes*). We observed specific, multisystemic, late-onset phenotypes, closely resembling the patient's syndrome, prominently in the facial skeleton and in neuronal tissue. Molecularly, we detected reduced levels of *N*-glycans in medaka and in the patient's fibroblasts. This hypo-*N*-glycosylation prominently affected protein abundance. Proteins of the basic glycosylation and glycoprotein-processing machinery were over-represented in a compensatory response, highlighting the regulatory topology of the network. Proteins of the retinal phototransduction machinery, conversely, were massively under-represented in the *alg2* model. These deficiencies relate to a specific failure to maintain rod photoreceptors, resulting in retinitis pigmentosa characterized by the progressive loss of these photoreceptors. Our work has explored only the tip of the iceberg of *N*-glycosylation-sensitive proteins, the function of which specifically impacts on cells, tissues and organs. Taking advantage of the well-described human mutation has allowed the complex interplay of *N*-glycosylated proteins and their contribution to development and disease to be addressed.

## Abstract (precis)

**Summary:** A fully rescuable, patient-based hypo-N-glycosylation ALG2 medaka model recapitulates the patient's multisystemic phenotypes, prominently in skeleton and neuronal tissue, allowing the cell type-specific impact of N-glycosylation to be investigated.

## INTRODUCTION

Protein glycosylation is a crucial prerequisite for proper protein function during the development and maintenance of differentiated cells, tissues, organs and entire organisms. This co- and post-translational protein modification is indispensable for correct protein folding and stability and thus immediately impacts on protein function. Strikingly, glycosylation and function of an enormous number of glycosylated target proteins is controlled by a comparably small number of proteins that direct the three major routes for oligosaccharide decoration of nascent proteins in the endoplasmic reticulum (ER), namely *N*- and *O*-linked glycosylation as well as *C*-mannosylation. Classical genetic approaches, i.e. the elimination of individual genes to study the cellular and organismal consequences, are reaching their limits because most of the mutations introduced trigger severe pleiotropic effects and result in (early embryonic) lethality.

Given the essential role of glycosylation, viable phenotypes, of varying degrees, are usually the result of hypomorphic mutations. Congenital disorders of glycosylation (CDG) in humans result from glycosylation deficiencies that often lead to severe multisystemic phenotypes. Glycosylation deficiencies affect neuronal development, as reflected by defects in myelination and brain formation (Hennet and Cabalzar, 2015). Eye development and functions are also impaired, as apparent by developmental abnormalities such as coloboma, strabismus and cataract formation and the progressive neurodegenerative disease retinitis pigmentosa (Cantagrel et al., 2010; Morava et al., 2010).

A number of key steps are required for a functional glycosylation machinery. For *N*-linked glycosylation, the first oligosaccharides are assembled at the ER membrane, initially on the cytosolic face. During this lipid-linked oligosaccharide (LLO) synthesis, the sequential addition of *N*-acetylglucosamines (GlcNAc), mannoses (Man) and glucoses (Glc) to the LLO precursor is catalysed by discrete asparagine-linked glycosylation (ALG) enzymes (Breitling and Aebi, 2013). The alpha-1,3/1,6-mannosyltransferase (ALG2) catalyses the first branch of the oligosaccharide structure by adding two mannoses to the Man1GlcNAc2-PP-Dol (Li et al., 2018; Thiel et al., 2003). After flipping into the ER and addition of further monosaccharides by several other ALG enzymes, the mature Glc3Man9GlcNAc2-PP-Dol is transferred to an asparagine acceptor amino acid of the NXS/T (X not proline) motif on the nascent polypeptide (Breitling and Aebi, 2013). After passing quality control, only correctly folded glycoproteins eventually exit the ER to the Golgi, where the *N*-linked glycan may undergo further modifications to form hybrid and complex glycan structures (Helenius and Aebi, 2004). The ALG enzymes and the resulting glycan structures are evolutionarily highly conserved across eukaryotes, further underpinning their fundamental importance (Park and Zhang, 2011).

Because null alleles in the corresponding genes are often lethal, the majority of CDG patients suffer from hypomorphic mutations that result in reduced protein functionality. It is noteworthy that ALG2-CDG patients (formerly CDG-Ii) appear normal at birth, but subsequently develop prominent and progressing deficits in eye, brain and muscles. In particular, iris coloboma, neuronal symptoms such as hypomyelination and intractable seizures as well as mental retardation, and affected muscle functionality (myasthenic syndrome) have been described (Cossins et al., 2013; Monies et al., 2016; Thiel et al., 2003). In addition patients suffer from hepatomegaly and coagulopathy (Haeuptle and Hennet, 2009; Varki et al., 2019). ALG2 acts at the base of the *N*-glycosylation cascade and is consequently highly sensitive to any alterations impacting on its functionality. Most mutations result in its loss of function, explaining the low number of ALG2 patients (to date nine patients from five families have been reported) (Cossins et al., 2013; Monies et al., 2016; Thiel et al., 2003). While mammalian models (mouse) have been predominantly employed to study the function of glycosyltransferases and the developmental consequences of mutations therein, the crucial importance of glycosyltransferases likely resulted in prominent pre-implantation and early embryonic defects (Matthijs et al., 1998; Thiel et al., 2006). Interestingly, a mouse model based on a hypomorphic mutation characterized in a mannose phosphate isomerase (MPI) patient turned out to be viable (Sharma et al., 2014).

The extrauterine development of teleost models (zebrafish, *Danio rerio*; medaka, *Oryzias latipes*), which relies on maternally provided resources, allows the developmental consequences of reduced glycosylation to be examined in the organismal context *in vivo*. To experimentally deconvolute and address those developmental processes most sensitively depending on a functional Alg2, we have modelled the human *ALG2-CDG* in fish. We have used the CRISPR/Cas9 toolbox (Gutierrez-Triana et al., 2018; Stemmer et al., 2015) to introduce mutations at the site orthologous to the site of mutation in a human ALG2 index patient (Thiel et al., 2003). The resulting small animal model presented here (homozygous lethal at early juvenile stages) displayed prominent deficiencies in the facial skeleton and deficits in the development and maintenance of the cardiovascular system, as well as an apparent failure to maintain rod photoreceptors. It resembled further multiple aspects of the multisystemic phenotype described in the human ALG2-CDG patients. We demonstrated the hypomorphic nature of the mutant alleles introduced by glycan structure profiling in the *alg2* fish model as well as in human patient fibroblasts. Proteome analysis of homozygous alg2^p.G336*/p.G336*^ mutants uncovered a putative compensatory action, resulting in, among other observations, an over-representation of enzymes providing nucleotide sugars, the building blocks of glycans. Strikingly, the hypo-*N*-glycosylation affected some cells more than others. We detected a progressive elimination of rod cells in the retina paralleled by an altered abundance of glycoproteins directly implicated with photoreceptor function. In line with the proposed maternal contribution of Alg2, transient supply of human or medaka *alg2* mRNA efficiently rescued the broad phenotypic spectrum and restored viability.

## RESULTS

### Medaka *alg2* variants recapitulated the symptoms of ALG2-CDG

**Fig. 1.**
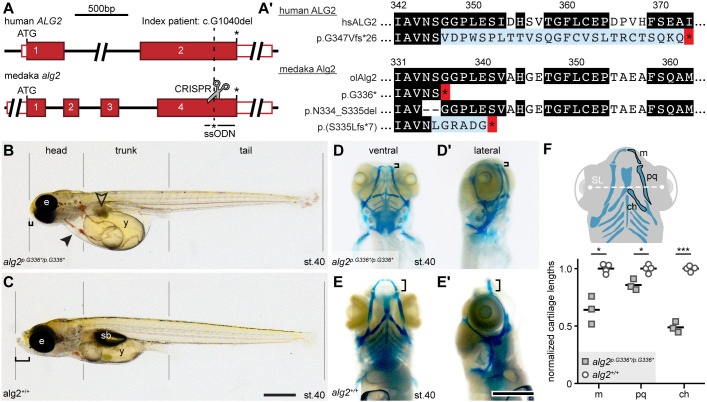
Homozygous medaka alg2^p.G336*/p.G336*^ mutants recapitulate ALG2-CDG multisystemic phenotype. (A) Schematic of human *ALG2* and the medaka orthologous *alg2* locus. The site of the ALG2 index patient mutation (c.1040delG) and targeted CRISPR genome editing (scissors) in medaka are indicated (dashed line). Single-stranded oligodeoxynucleotide (ssODN) donor for introduction of premature STOP (asterisk) is given. Red box, coding exon; white box, untranslated region. (A′) Predicted protein variants at the given position of human ALG2 index patient and corresponding sites of the stable mutant fish lines. Black, highly conserved amino acids (AA); blue, different AA resulting from underlying frame shift; red, premature STOP. Nomenclature of protein variants according to den Dunnen et al. (2016). For AA and genomic sequences of medaka *alg2* mutant alleles, see Fig. S1C. (B,C) Representative phenotype of alg2^p.G336*/p.G336*^ mutant (B) and corresponding wt (C) sibling at stage 40. Note in the mutant craniofacial defects, including prominently shortened snout (bracket), persistent yolk sac (y), non-inflated swim bladder (sb), enlarged liver (unfilled arrowhead), secondary tubular heart (black arrowhead) and clogging of blood, slightly smaller eyes (e). (D-E′) Alcian Blue staining of alg2^p.G336*/p.G336*^ mutant (D,D′) and *alg2*^+/+^ (E,E′) embryos at stage 40 reveal dramatic reduction of the craniofacial cartilages (brackets). Ventral view in D,E; lateral view D′,E′. (F) Top: Schematic (ventral view) of craniofacial cartilages. Bottom: Comparison of measured cartilage lengths (m, Meckel's cartilage; pq, palatoquadrate; ch, ceratohyal) normalized to standard length (SL, distance between the lenses) reveals significant reduction of all three cartilage structures in alg2^p.G336/p.G336*^ mutants (*n*=3) compared with *alg2^+/+^* (*n*=4; two-tailed nonparametric Student's *t*-test; *P<0.05; ***P<0.001). Scale bars: 0.5 mm.

Because the complete loss of function of many glycosylation-related proteins results in early embryonic lethality, we aimed to introduce a putative human hypomorphic mutation into the medaka genome. We took advantage of the described hypomorphic mutation in the human ALG2 index patient (maternal allele c.1040delG that translates to p.G347Vfs*26; Fig. 1A,A′; Thiel et al., 2003) and introduced mutations at the corresponding position of the evolutionarily highly conserved orthologous medaka *alg2* gene (Fig. 1A,A′, Fig. S1A-C). Employing targeted CRISPR/Cas9 genome editing (Fig. 1A, Fig. S1C), we introduced the human mutation by single-stranded oligodeoxynucleotide (ssODN) integration. The non-homologous end-joining mechanism triggered by the CRISPR/Cas9 DNA cleavage yields several different alleles in parallel to the desired integration of the ssODN. We thus used survival of the genome-edited embryos to select for viable Alg2 variants in medaka whether ssODN or insertion/deletion based. We established three different alleles in stable fish lines (predicted resulting proteins are depicted in Fig. 1A′; see Fig. S1C for genomic sequences) that show alterations corresponding to the site of mutation in the ALG2 index patient (Fig. 1A, dashed line; Thiel et al., 2003). The ssODN-edited allele displayed an early premature STOP codon (XM_004077863:c.1006-1011delinsTAAGG that translates to XP_004077911:p.G336*). A second allele represented an in-frame deletion, lacking six nucleotides at the C terminus (XM_004077863:c.999-1004del that translates to XP_004077911:p.N334_S335del). A third allele harboured a large deletion at the C terminus [XM_004077863:c.1002_1218+5delinsTCTG that translates to XP_004077911:p.(S335Lfs*8)]. Under homozygosity, all three alleles displayed fully comparable phenotypes and were lethal at early juvenile stages. For the remainder of the study, we focused on the precisely edited p.G336* variant.

**Fig. 2.**
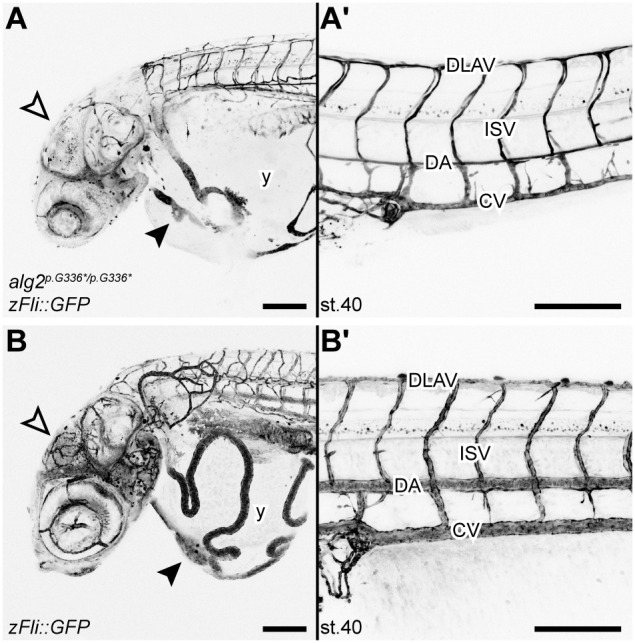
alg2^p.G336*/p.G336*^ mutant embryos display severe alterations in vasculature anatomy. (A-B′) Homozygous alg2^p.G336*/p.G336*^ allele in *zFli::GFP* reporter line (stage 40) showed an overall underdeveloped and thin vasculature anatomy (A) compared with unaffected stage-matched *zFli::GFP* siblings (B). Branching of cranial vessels (unfilled arrowheads) and developing gill vasculature were missing entirely in the homozygous mutant embryos. Main vessels in the yolk (y) were shortened and thin as were all vessels of the trunk and tail in mutants (A′) compared with control (B′). Note the very small atrium in mutants compared with controls (black arrowheads). CV, caudal vein; DA, dorsal aorta; DLAV, dorsal longitudinal anastomose vessel; ISV, intersegmental vessel. Scale bars: 200 µm.

Interestingly, early embryonic development of the mutants was indistinguishable from that of wild type (wt) and changes in gross morphology only became apparent just prior to hatching at stage 39 (Iwamatsu, 2004). Similar to the multisystemic effects reported for ALG2-CDG patients, we observed matching multisystemic phenotypes with a late onset during embryonic development that progressed quickly (Fig. 1B). The homozygous mutant embryos failed to engulf the yolk and a prominent yolk sac was still apparent after hatching. Already at late embryonic stages the liver was enlarged compared with age-matched wt siblings (Fig. 1B, unfilled arrowhead). The air-inflated swim bladder seen in wt (*alg2^+/+^*) hatchlings (Fig. 1C) was not detectable in the mutants. In contrast to the overall comparable size of the head, trunk and tail, the eyes were slightly smaller and prominent craniofacial dysmorphisms resulted in a severely shortened snout (Fig. 1B,D, black bracket). The reduction in jaw size was caused by significantly shorter Meckel's cartilage (*P*=0.02), palatoquadrate (*P*=0.03) and ceratohyal (*P*<0.001) cartilage compared with stage-matched *alg2^+/+^* siblings (Fig. 1D-F). The multisystemic phenotypes resulted in lethality of homozygous mutants at 2-3 days post-hatching (dph). Thus, an endpoint genotyping analysis at 4 dph of batches collected from alg2^p.G336*/+^ in-crosses revealed that the survivors were either heterozygous (alg2^p.G336*/+^; *n*=31) or wt (*alg2^+/+^*; *n*=20) and no homozygous alg2^p.G336*/p.G336*^ hatchlings were detected (Table S1). The observed dramatic reduction of blood flow (from stage 39 onwards), followed by blood clogging and eventually a complete arrest of blood circulation can be attributed to a progressive loss of heart function. The initially well-formed two-chambered heart thinned out and became secondarily tubular (Fig. 1B, black arrowhead). Similarly, the major vessels were thin and oedema formed around the heart and the eyes. The overall vasculature anatomy was underdeveloped in homozygous alg2^p.G336*/p.G336/^ mutant embryos (Fig. 2A,A′), which was most obvious by a lack of cranial and developing gill vessels as well as thin overall vasculature compared with unaffected *zFli*::*GFP* reporter siblings at stage 40 (Fig. 2B,B′). In a representative case, Haematoxylin and Eosin (H&E) staining performed on transverse sections of stage 40 alg2^p.G336*/p.G336*^ mutant and *alg2*^+/+^ control hatchlings revealed neuronal abnormalities reflected by reduced white matter in the mid- and hindbrain as well as in the optic tectum of mutants (Fig. S2), a phenotype that has been described for ALG2 index patients. Interestingly, among the clutch of homozygous alg2^p.G336*/p.G336*^ mutants, the interindividual variance in the phenotype was minute.

Taken together, the medaka model presented here strikingly recapitulated morphological symptoms described for ALG2-CDG. Because hypoglycosylation was reported for human CDG patients (Thiel et al., 2003), we compared the state of glycosylation in the medaka model and human ALG2 index patient fibroblasts.

### Medaka Alg2:p.G336* variant resulted in hypo-*N*-glycosylation

**Fig. 3.**
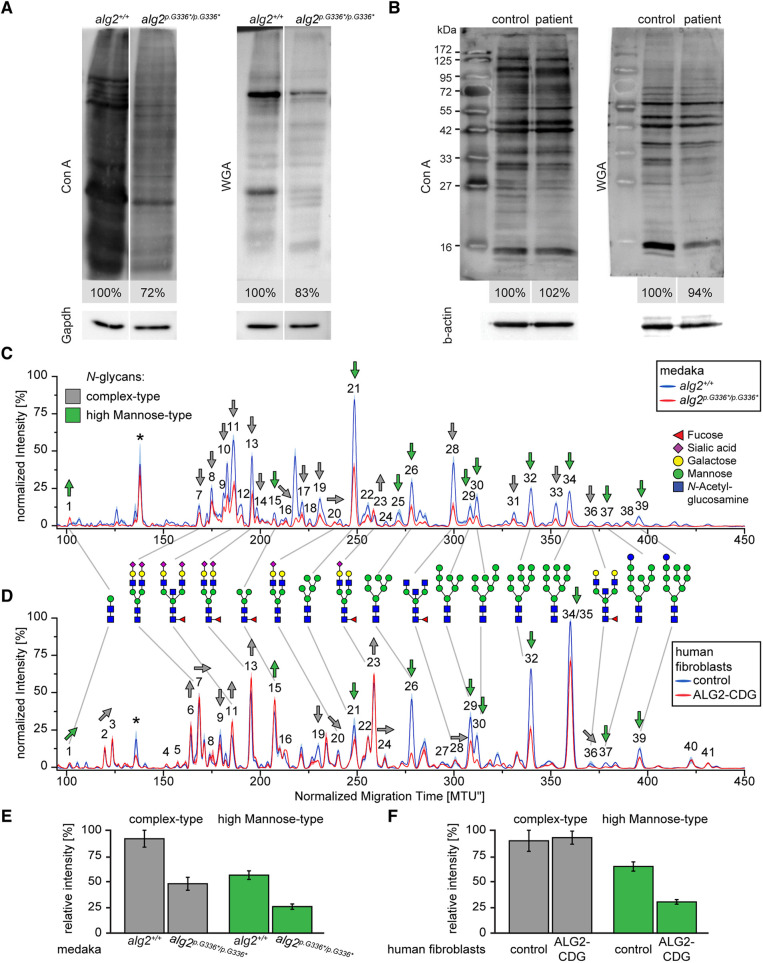
Medaka Alg2:p.G336* and human ALG2:p.G347Vfs*26 are hypomorphic variants. (A,B) Lectin blots on total protein lysates of medaka *alg2^+/+^* and homozygous alg2^p.G336*/p.G336*^ mutant embryos (A) as well as human control and ALG2 CDG-patient fibroblasts (B). Intensity values normalized to internal loading control (Gapdh/β-actin) and wt or control samples. Con A, concanavalin A; WGA, wheat germ agglutinin. (C,D) xCGE-LIF-generated *N*-glycan fingerprints comparing medaka *alg2^+/+^* (blue) with homozygous alg2^p.G336*/p.G336*^ mutant embryos (red; C) and human control (blue) with ALG2 index patient (red; D) fibroblasts. *N*-glycan fingerprints represent average values from three biological replicates with the standard deviation shown as semi-transparent band. Asterisks indicate standard for migration time (MTU) normalization. Trends in observed quantitative changes for individual *N*-glycan structures are indicated by grey (complex-type) and green (high mannose-type) arrows with the direction indicating up- or downregulation. Peak numbers refer to identified *N*-glycan structures (see Table S2 for a list of all identified *N*-glycans). (E,F) Quantification of complex type (grey) and high mannose-type (green) *N*-glycan structures in medaka wt versus mutant (E) and control versus patient fibroblasts (F) from three biological replicates. Intensities represent the sum of all peak heights of complex- and high mannose-type *N*-glycan normalized to 100% of maximum for each chart, respectively.

To address the global levels of protein *N*-glycosylation, we took advantage of the specific glycan-binding capacity of the lectin concanavalin A (Con A). We used Con A to detect mannose and wheat germ agglutinin (WGA) to detect *N*-acetylglucosamine. Lectin blots on proteins extracted from alg2^p.G336*/p.G336*^ mutant medaka hatchlings (stage 40) showed reduced levels of mannose (72%, ConA) and *N*-acetylglucosamine (83%; WGA) compared with *alg2^+/+^* siblings (Fig. 3A). The analysis of fibroblasts derived from the ALG2 index patient by the same approach did not show this tendency as the levels of mannose (102%; Con A) and *N*-acetylglucosamine (94%; WGA blot) were comparable (Fig. 3B).

To investigate further how the medaka alg2^p.G336*/p.G336*^ mutant and fibroblasts of the ALG2 index patient affected the *N*-glycan fingerprint, we performed an extended *N*-glycan analysis by multiplexed capillary gel electrophoresis with laser-induced fluorescence detection (xCGE-LIF). This approach enables the identification and relative quantification of individual *N*-glycan structures via normalization to an internal standard (Fig. S3), allowing direct quantitative comparison between samples of different origin such as fish and human (Hennig et al., 2016). Comparing the *N*-glycan fingerprints of wt Alg2 medaka (Fig. 3C, blue) with the Alg2:p.G366* *N*-glycan fingerprints (Fig. 3C, red) severe hypo-*N*-glycosylation was apparent by a reduction in the levels of complex-type (48% less) and high-mannose-type (54% less) *N*-glycans (Fig. 3E). This hypo-*N*-glycosylation affected all structures to a similar extent with only minor qualitative differences.

The *N*-glycan fingerprint of the human fibroblast control samples (Fig. 3D, blue) revealed a high structural similarity to the medaka *N*-glycome reflecting the high degree of evolutionary conservation of the glycosylation machinery. Fibroblasts of the human ALG2 index patient (Fig. 3D, red) showed no substantial changes of the complex-type *N*-glycosylation compared with the control samples (Fig. 3D, blue). In sharp contrast, the abundance of high-mannose-type *N*-glycans was decreased by 53% (Fig. 3F). This resulted in an overall reduction in *N*-glycosylation of 19% in the patient-derived fibroblasts, reflecting the described hypo-*N*-glycosylation in these cells.

Our analysis revealed that both the human ALG2:p.G347Vfs*26 protein and the medaka mutants homozygously expressing the Alg2:p.G336* protein resulted in prominent hypo-*N*-glycosylation. Because glycosylation is known to affect protein stability, we investigated the effects of the hypo-*N*-glycosylation on the proteome composition in homozygous alg2^p.G336*/p.G336*^ medaka mutants.

### Hypo-*N*-glycosylation altered the abundance of proteins involved in the glycosylation machinery and the phototransduction pathway

**Fig. 4.**
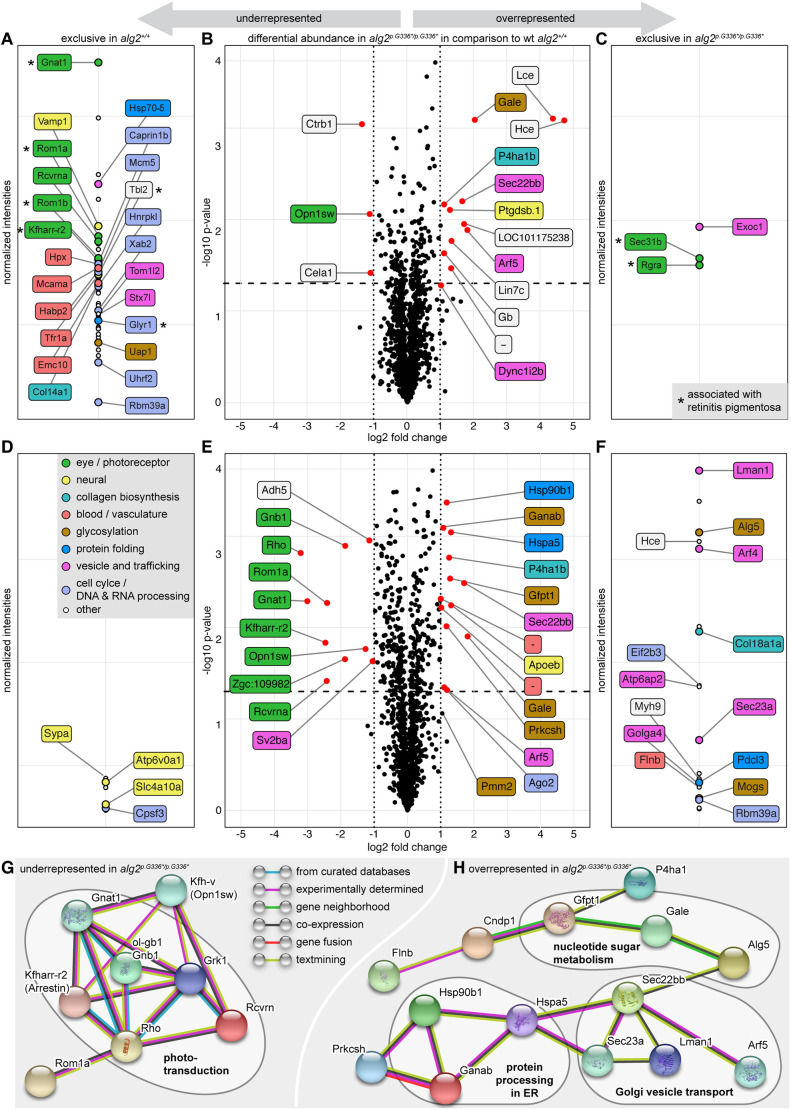
alg2^p.G336*/p.G336*^ mutants show reduced levels of proteins involved in phototransduction but higher abundance of the glycosylation machinery. (A-F) Unbiased mass spectrometry of chemically labelled peptides derived from total protein lysates of deyolked wild-type (wt) *alg2^+/+^* and alg2^p.G336*/p.G336*^ whole hatchlings (A-C) and enucleated eyes (D-F) at stage 40. Differentially regulated proteins are shown in volcano plots (B-E), proteins exclusively detected in wild-type or mutants are presented in intensity plots (A,C,D,F). Coloured labelling and grouping according to (predicted) protein function retrieved by text-mining. (A,D) Intensity plots of proteins exclusively detected in wt samples amounted to 56 proteins in whole hatchling samples (A) and to seven proteins in the enucleated eye samples (D). (B,E) Volcano plots of protein abundance comparison between *alg2^+/+^* and alg2^p.G336*/p.G336*^ reveals no major regulation in protein abundance, yet distinct changes. Proteins with significant (*P*<0.05, dashed line) and more than 2-fold change (dotted lines) are indicated (red dots) and labelled. (C,F) Intensity plot of proteins exclusively detected in alg2^p.G336*/p.G336*^ mutant samples amounted to three proteins for the whole hatchling samples (C) and 19 proteins in the enucleated eye samples (F). (G,H) Network analysis of functional associations and/or interactions of all regulated and exclusive proteins in eye samples. Different clusters of regulated proteins were identified using STRING software (medium confidence score, disconnected nodes hidden; Szklarczyk et al., 2019). Among the under-represented proteins in the mutants, photoreceptor-related players of the phototransduction pathway were enriched (G). Among the overrepresented ones, proteins involved in protein processing in the ER and of the nucleotide sugar metabolism pathways were pronounced (H). The coloured lines linking different proteins represent the types of evidence (blue: from curated databases; purple: experimentally determined; green: gene neighbourhood; black: co-expression; red: gene fusion; light green: text-mining). For full proteomics data, see Table S3. Asterisks in A and C indicate proteins associated with retinitis pigmentosa. Total protein extracts were derived from three replicates each comprising six stage 40, de-yolked hatchlings per genotype; analysis on eye samples were conducted on four replicates with 30 enucleated eyes of stage 40 hatchlings per genotype, respectively.

To address potential changes in the proteome, we performed an unbiased proteomics analysis. We analysed total protein extracts of homozygous alg2^p.G336*/p.G336*^ and *alg2^+/+^* hatchlings (stage 40, de-yolked, three replicates with six hatchlings each) upon isotopic labelling by dimethylation at the level of the peptides (Boersema et al., 2009). Interestingly, the differential proteome analysis did reveal very distinct differences, but no global regulation of protein levels. Analysis of the proteomes of alg2^p.G336*/p.G336*^ mutant fish (Fig. 4A-C) uncovered 15 proteins with a significant (*P*<0.05) differential abundance differing more than 2-fold (Fig. 4B) in comparison with wt controls.

Among the 12 proteins over-represented in the alg2^p.G336*/p.G336*^ mutant fish (Fig. 4B), we identified the secreted metalloendopeptidases low (lce) and high (hce) choriolytic enzymes (19- and 22-fold upregulation, respectively) as the top hits followed by UDP-glucose 4-epimerase (Gale). The latter catalyses two distinct interconverting reactions: UDP-galactose (UDP-Gal) from and to UDP-Glc as well as *N*-acetylgalactosamine (GalNAc) from and to GlcNAc (Daenzer et al., 2012) of which all four nucleotide sugars serve as substrates for glycan synthesis. The remaining over-represented proteins are related to endopeptidases or play a role in vesicle transport, haem binding or collagen synthesis.

The three under-represented proteins were a putative violet-sensitive opsin and two proteins belonging to the peptidase S1 family (Fig. 4B). In addition, a larger number of proteins were present in either wt or mutant samples only (Table S3). These exclusive hits amounted to 64 in *alg2*^+/+^ (Fig. 4A) and three in the mutant alg2^p.G336*/p.G336*^ samples (Fig. 4C). Those proteins can be grouped into classes involved in vesicle trafficking, regulation of the blood and circulatory system, and protein folding, as well as proteins with described and predicted functionality in neuronal and especially eye and photoreceptor-related activity. Interestingly, and consistent with a role of ALG2 in the development of the myasthenic syndrome, a fatigable muscle weakness (Cossins et al., 2013), Slc25a1 and Vamp1 were absent from the mutant proteome. Strikingly, eight of the 67 exclusive candidates are associated with retinitis pigmentosa (human orthologues in parentheses): Gnat1 (GNAT1), Rom1a and Rom1b (ROM1), Kfharr-r2 (SAG, ARRESTIN, RP47), Rgra (RGR, RP44), Tbl2 (TBL2), Sec31b (SEC31B) and Glyr1 (GLYR1) (Ba-Abbad et al., 2018; Carrigan et al., 2016; Conley et al., 2017; Sullivan et al., 2017).

To refine the relative abundance of eye-related proteins, we performed a second unbiased proteomics experiment on enucleated eyes (four replicates, 30 eyes each) of alg2^p.G336*/p.G336*^ and *alg2*^+/+^ hatchlings at stage 40. This analysis identified 23 differentially expressed proteins (*P*<0.05) with more than 2-fold change and 26 proteins exclusively found in wt or mutant samples (Fig. 4D-F); again, global protein levels were not changed. A protein-protein interaction analysis (STRING software v11.0; Szklarczyk et al., 2019) on the regulated and exclusively found proteins revealed distinct networks affected by hypo-*N*-glycosylation. For the proteins that were under-represented in the alg2^p.G336*/p.G336*^ mutants, players of the phototransduction pathway active in photoreceptor cells were pronounced (6/35 proteins described in KEGG pathway ola04744, false discovery rate 3.00×10^−1^; Fig. 4G). For the over-represented proteins, the eye samples showed the same trend as the whole organism, i.e. higher abundance of enzymes of the glycosylation machinery involving protein processing in the ER and Golgi vesicle transport (5/155 proteins described in KEGG pathway ola04141, false discovery rate 2.46×10^−1^) as well as nucleotide sugar metabolism (Fig. 4H).

In addition to the consistent over-representation of Gale, we detected over-representation of Glutamine-fructose-6-phosphate transaminase 1 (Gfpt1), which catalyses the formation of glucosamine-6-phosphate, a precursor for UDP-GlcNAc (Oki et al., 1999); Dolichyl-phosphate beta-glucosyltransferase (Alg5), which provides Dol-P-Glc; and Phosphomannomutase 2 (Pmm2, *P*=0.087), which catalyses the conversion of Man-6-P to Man-1-P as a precursor for Dol-P-Man. ER-resident enzymes involved in core *N*-glycan processing to trim the glucoses in the α1,3-arm were also well represented (Zuber et al., 2000): the first glucose removal is catalysed by Mannosyl-oligosaccharide glucosidase (Mogs), followed by the Glucosidase II alpha subunit (Ganab) and Protein kinase c substrate 80K-H (Prkcsh), which trim the next two glucoses (Grinna and Robbins, 1979; Burns and Touster, 1982; Grinna and Robbins, 1980). Members of the subsequent protein folding control (Hspa5 and Hsp90b1) as well as the ER-to-Golgi transport system were over-represented in addition (Lman1, Sec23a, Sec22bb; Khoriaty et al., 2012).

Taken together, the molecular phenotypes detected in the proteomics analysis reinforced the morphologically apparent phenotypes and uncovered the mis-regulation of factors known for blood circulation and blood vessel formation in alg2^p.G336*/p.G336*^ mutants. In addition, this analysis pointed towards a compensatory over-representation of parts of the *N*-glycosylation pathway (Fig. 4H). It also revealed a massively affected phototransduction machinery (Fig. 4G) with potential impact on the function of the retina, which we decided to investigate further.

### Loss of rod photoreceptors caused by alg2^p.G336*^-mediated hypo-*N*-glycosylation

**Fig. 5.**
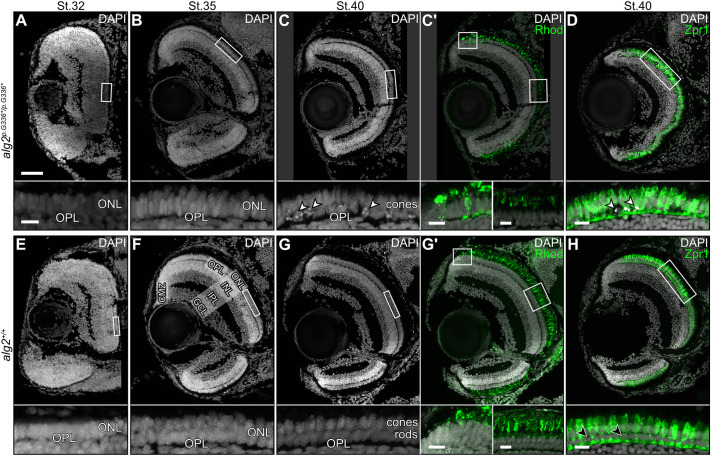
Rod photoreceptors are specifically affected in alg2^p.G336*/p.G336*^ mutant retinae. (A-H) Time series of retinal development as depicted by DAPI and immunohistochemistry staining against rod (Rhodopsin) and cone (Zpr1) specific markers on cryotome sections in homozygous alg2^p.G336*/p.G336*^ (A-D) and *alg2^+/+^* (E-H) siblings at stage 32 (A,E), stage 35 (B,F) and hatching stage 40 (C-D,G-H). Separation of rod and cone photoreceptors from stage 35 onwards (F,G) failed in alg2^p.G336*/p.G336*^ mutant embryos (B,C). The inner row of the alg2^p.G336*/p.G336*^ ONL harbours pyknotic nuclei (C-D, white arrowheads) at the position of rod nuclei in wild-type embryos (G-H, black arrowheads). Zpr1 staining is unaffected in alg2^p.G336*/p.G336*^ mutant embryos but appears irregular due to affected rod cells. CMZ, ciliary marginal zone; GCL, ganglion cell layer; INL, inner nuclear layer; IPL, inner plexiform layer; ONL, outer nuclear layer; OPL, outer plexiform layer. Insets show magnified views of the boxed areas in the panels above. Scale bars: 50 µm (main panels); 10 µm (insets). Six specimens per genotype and stage were used for analysis.

To investigate the developmental impact on neuronal structures, we analysed retinal development at different developmental stages (six specimens per stage and genotype) on DAPI-stained histological sections of alg2^p.G336*/p.G336*^ in comparison with *alg2^+/+^* embryos (Fig. 5). At the onset of retinal lamination (stage 32), eyes were indistinguishable between the different genotypes (Fig. 5A,E). At stage 35, alg2^p.G336*/p.G336*^ mutants exhibited an apparent difference in the outer nuclear layer (ONL), where rod and cone photoreceptors did not segregate but rather extended throughout the entire ONL (Fig. 5B). In wt siblings, by contrast, the cone and rod photoreceptors had already started to separate (Fig. 5F) resulting in bi-layering with the nuclei of cones prominently confined to the outer row and the nuclei of rods remaining in an inner row underneath. At hatching stage (stage 40), the retinae of both genotypes appeared normal and well laminated, but the nuclei in the inner row of photoreceptors were pyknotic in the mutants (Fig. 5C, arrowheads), a situation never observed in the wt siblings (Fig. 5G). We investigated the molecular identity of these photoreceptors by immunofluorescence using the rod cell marker Rhodopsin (Fig. 5C′,G′, Fig. S4A-D) and the cone cell-specific marker Zpr1 (Fig. 5D,H, Fig. S4E-H). In contrast to wt retinae, Rhodopsin staining in the alg2^p.G336*/p.G336*^ mutants was not uniform (Fig. 5C′) and the outer segments of the rod cells did not extend as far into the retinal pigment epithelium as those of wt siblings (Fig. 5G′). The cone photoreceptors (detected by Zpr1 staining), in contrast, were seemingly unaffected by the hypo-*N*-glycosylation condition (Fig. 5D) in the alg2^p.G336*/p.G336*^ mutants.

**Fig. 6.**
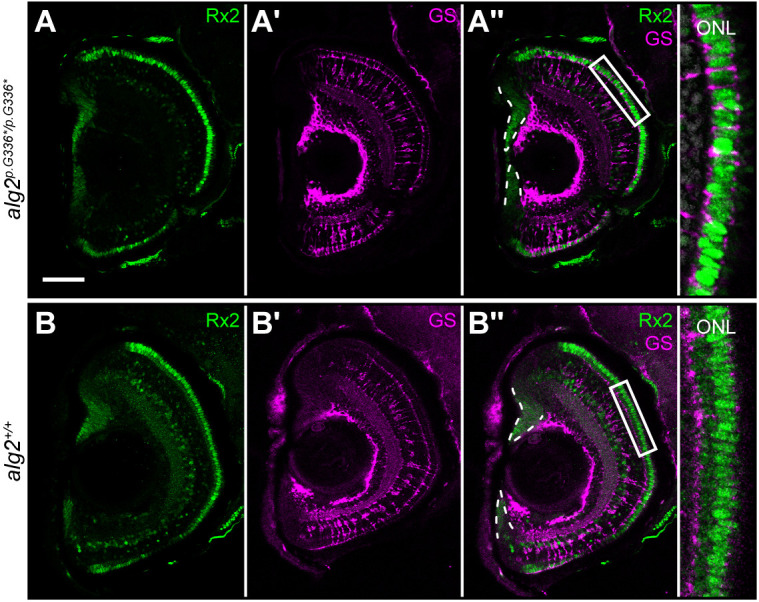
Sources of photoreceptor differentiation and regeneration are unaffected in alg2^p.G336*/p.G336*^ hatchlings. Transverse cryotome sections through the eyes of stage 40 alg2^p.G336*/p.G336*^ (A-A″) and *alg2*^+/+^ (B-B″) hatchlings. Immunohistochemistry detecting Rx2 (green) as a marker for retinal stem and progenitor cells, Müller glia (MG) cells and photoreceptors, and glutamine synthetase (GS, magenta) highlighting MG cell bodies did not reveal alterations between alg2^p.G336*/p.G336*^ (A-A″) and wt controls (B-B″) in the CMZ nor the central retina except for the outer nuclear layer (insets). Here, Rx2 cone photoreceptor staining reconfirmed the monolayer of photoreceptors in alg2^p.G336*/p.G336*^ hatchlings in contrast to the *alg2^+/+^* bi-layer (compare with Fig. 5C,G). Scale bar: 50 µm. Insets show magnifications of boxed areas. Four specimens per genotype were used for analysis.

As only the rod photoreceptors were affected in alg2^p.G336*/p.G336*^ mutant retinae, we investigated whether this was caused by a failure in their development and differentiation or their maintenance. Whereas stem and progenitor cells that reside in the ciliary marginal zone (CMZ) build the growing retina (including all photoreceptors; Centanin et al., 2014), Müller glia (MG) are triggered to divide and compensate for the acute loss of photoreceptors (Lust and Wittbrodt, 2018). We employed the retina-specific homeobox gene *Rx2* as marker for photoreceptors, stem and progenitor cells in the CMZ and MG cells (Reinhardt et al., 2015) in alg2^p.G336*/p.G336*^ and *alg2^+/+^* hatchlings (Fig. 6). Immunohistochemistry employing Rx2-specific antibodies revealed a severe reduction of the inner layer of (rod) photoreceptor cells in the alg2^p.G336*/p.G336*^ mutant retinae (Fig. 6A, right-hand panel) compared with the photoreceptor bi-layer in wt hatchlings (Fig. 6B, right-hand panel). This is consistent with DAPI analysis (Fig. 5) highlighting defects in lamination of the outer nuclear layer in alg2^p.G336*/p.G336*^ mutants (Fig. 5A). The shape of the CMZ and the abundance of MG cells (anti-glutamine synthetase staining), in contrast, were comparable between alg2^p.G336*/p.G336*^ (Fig. 6A′-A″) and *alg2^+/+^* (Fig. 6B′-B″) siblings, indicating proper proliferation and differentiation of the growing retina.

**Fig. 7.**
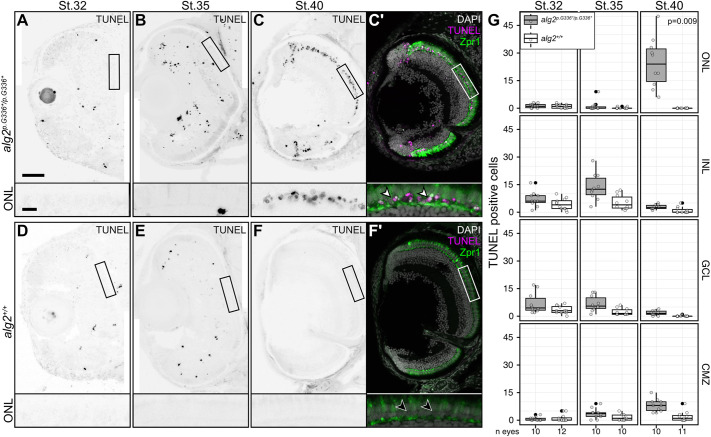
Hypo-*N*-glycosylation specifically triggers loss of rod photoreceptors in alg2^p.G336*/p.G336*^ hatchlings. (A-F′) Transverse cryotome sections through the eyes of stage 32 (A,D), stage 35 (B,E) and hatching stage 40 (C,C′,F,F′) of alg2^p.G336*/p.G336*^ (A-C′) and *alg2^+/+^* (D-F′) hatchlings stained for apoptotic cells (TUNEL), cone photoreceptors (Zpr1) and nuclei (DAPI). Enrichment of TUNEL-positive cells in the outer nuclear layer of stage 40 hatchlings (ONL; white arrowheads) overlays with the location of rod photoreceptors (black arrowheads). (G) Quantification of TUNEL-positive cells on central eye sections. Highly significant enrichment of TUNEL-positive cells was observed in the ONL of alg2^p.G336*/p.G336*^ hatchlings compared with stage-matched *alg2^+/+^* siblings (*P*=0.009). All other layers and stages displayed slight but not significant enrichments of apoptotic cells in alg2^p.G336*/p.G336*^ versus *alg2^+/+^* samples (pairwise wilcoxon rank sum tests). Box plots depict median (thick black horizontal line); the extremes of the box are the 25th and 75th percentiles; the whiskers represent the minimum and maximum outliers; the filled circles represent extreme outliers. Non-filled scattered circles indicate raw data. CMZ, ciliary marginal zone; GCL, ganglion cell layer; INL, inner nuclear layer; IPL, inner plexiform layer; OPL, outer plexiform layer. Scale bars: 50 µm (main panels); 10 µm (insets). Eyes of six specimens per genotype and stage were used for analysis; non-central sections were excluded from analysis.

To determine whether rod photoreceptor cells in alg2^p.G336*/p.G336*^ mutants were actively eliminated, (programmed) cell death was visualized by terminal deoxynucleotidyl transferase dUTP nick end labelling (TUNEL) staining in a developmental series. In mutant embryos, apoptotic cells were sparsely scattered throughout the entire retina during development (Fig. 7A,B) with numbers only slightly but not significantly increased in comparison with wt siblings (Fig. 7D,E). In strong contrast to earlier stages, TUNEL-positive cells were strongly and highly significantly increased in the photoreceptor layer at stage 40 (Fig. 7C,C′,G) whereas stage-matched wt siblings showed very low numbers of apoptotic cells in the retina (Fig. 7F-G) and none in the ONL. This finding indicates that rod photoreceptors were initially formed during development and post-embryonic growth of the retina, but were not maintained and were subsequently eliminated by apoptosis. This loss of rod photoreceptors is characteristic for retinitis pigmentosa or night blindness.

### alg2^p.G336*/p.G336*^ mutants were rescued by exogenous supply of full-length *alg2* mRNAs

**Fig. 8.**
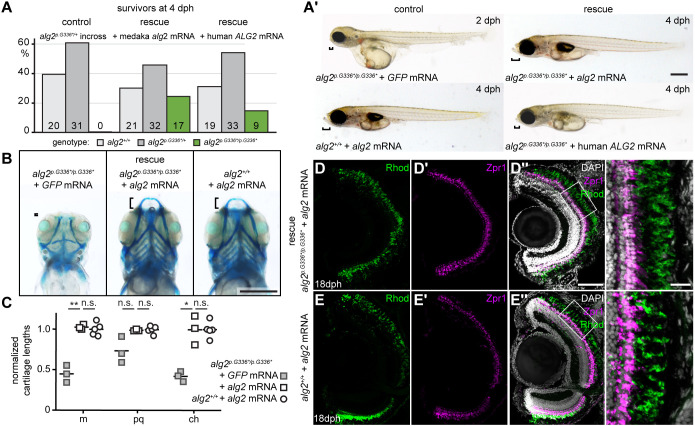
Human and medaka *alg2* mRNA rescue the multisystemic phenotype in alg2^p.G336*/p.G336*^. (A) Individual genotyping of hatchlings at 4 days post hatching (dph) of batches descending from heterozygous alg2^p.G336*/+^ crosses revealed the absence of homozygous survivors. Rescue with medaka or human full-length *alg2* mRNA restored their survival close to Mendelian distribution (see also Table S1). Number of hatchlings is shown at the base of the bars. (A′) The multisystemic phenotype of alg2^p.G336*/p.G336*^ hatchlings were fully rescued by medaka *alg2* mRNA but not *GFP* mRNA control injections at the one-cell stage. Exogenous supply of *alg2* mRNA in *alg2^+/+^* siblings did not result in any phenotype. Full-length human *ALG2* mRNA rescued the gross morphology phenotype, but to a lesser extent, evident in the partially rescued snout size (brackets). (B) Alcian Blue staining (ventral view, stage 40) of *GFP* mRNA control (left) and full-length medaka *alg2* mRNA rescue (central) injected at the one-cell stage into alg2^p.G336*/p.G336*^ zygotes as well as full-length medaka *alg2* mRNA injected into *alg2^+/+^* (right) specimens. Cartilage anatomy in rescued and exogenous supplied *alg2* in wt was indistinguishable. (C) Quantitative analysis of cartilage lengths, normalized to standard length (SL, distance between lenses; see Fig. 1F). Full-length *alg2* mRNA completely rescues craniofacial cartilage structures in contrast to control *GFP* mRNA injections. Exogenous *alg2* mRNA does not alter cartilage structures. m, Meckel's cartilage; pq, palatoquadrate; ch, ceratohyal. *P<0.05; ***P<0.001 (two-tailed nonparametric Student's *t*-test). n.s., not significant. Black lines indicate the mean. (D-E″) Immunohistochemistry staining against the photoreceptor-specific markers Rhodopsin (Rhod, rod photoreceptors, green) and Zpr1 (cone photoreceptors, magenta) and DAPI on transverse cryotome sections through the eyes of stage 40 alg2^p.G336*/p.G336*^ and *alg2^+/+^* hatchlings rescued after injection with full-length *alg2* mRNA at the one-cell stage. Right-hand panels show magnifications of the boxed areas. Scale bars: 0.5 mm (A′,B); 50 µm (D″); 10 µm (right-hand panels in D-E″).

Given that early embryogenesis was unaffected in the alg2^p.G336*/p.G336*^ mutants, we reasoned that the onset of the phenotype is caused by a depletion of maternally contributed Alg2. Exogenously supplied wt Alg2 was therefore expected to rescue and delay the onset of the phenotype. To address the rescue potential of *alg2* mRNA, we microinjected full-length medaka *alg2* (100-200 ng/µl) or human *ALG2* (33-50 ng/µl) mRNA at the one-cell stage. We performed an endpoint genotyping analysis at 4 dph of control and *alg2* mRNA-injected batches descending from alg2^p.G336*/+^ crosses (Fig. 8A, Table S1). Individual genotyping of the uninjected control embryos revealed only wt and heterozygous embryos; homozygous alg2^p.G336*/p.G336*^ mutants were absent. Upon injection of medaka or human *alg2* mRNA, the survival of homozygous mutant embryos was restored to a Mendelian distribution (Fig. 8A, Table S1). In addition, either *alg2* mRNA efficiently rescued the gross morphology phenotype and expanded the life-span by at least 16 days. Gross morphology was fully restored in injected alg2^p.G336*/p.G336*^ and genotyped mutant embryos (medaka *alg2*, *n*=28; human *ALG2*, *n*=11) whereas control injection with *GFP* mRNA (100-200 ng/µl) had no rescue effect (*n*=4; individually genotyped alg2^p.G336*/p.G336*^ homozygotes; Fig. 8A′). The exogenous supply of medaka *alg2* mRNA did not lead to apparent phenotypes in either heterozygous alg2^p.G336*/+^ (*n*=54) or *alg2^+/+^* siblings (*n*=36; Fig. 8A′). Alcian Blue staining of injected embryos (alg2^p.G336*/p.G336*^+*GFP* mRNA, *n*=3; alg2^p.G336*/p.G336*^+*alg2* mRNA, *n*=3; *alg2^+/+^*+*alg2* mRNA, *n*=5; Fig. 8B,C) showed a full reversion of the head cartilage phenotype in the rescued alg2^p.G336*/p.G336*^ mutant embryos (*P*=0.007, *P*=0.02, *P*=0.11 for Meckel's cartilage, ceratohyal and palatoquadrate cartilages, respectively; two-tailed nonparametric Student's *t*-test; Fig. 8C). Cartilage lengths were indistinguishable between the rescue injections and medaka *alg2* overexpression in *alg2^+/+^* embryos (*P*=0.48, *P*=0.97, *P*=0.61 for Meckel's cartilage, ceratohyal and palatoquadrate cartilages, respectively; two-tailed nonparametric Student's *t*-test; Fig. 8C). Injection of medaka *alg2* mRNA not only rescued gross morphology, but also restored viability (at least) until the fish were used for downstream analysis at 18 dph. The apparent failure of alg2^p.G336*/p.G336*^ hatchlings to maintain rod photoreceptors detailed above was efficiently rescued (*n*=4) as well by *alg2* mRNA injection. Analysis of rod and cone photoreceptors by immunohistochemistry on retinal cross-sections demonstrated the full restoration of the bi-layered structure of the ONL in the entire lining of the retina of rescued alg2^p.G336*/p.G336*^ hatchlings (Fig. 8D-D″), which were indistinguishable from *alg2^+/+^* siblings (Fig. 5G,H) or wt siblings additionally expressing injected medaka *alg2* mRNA (Fig. 8E-E″).

Taken together, the medaka alg2^p.G336*/p.G336*^ line presented in this study is a fully rescuable organismal model for hypo-*N*-glycosylation, recapitulating several clinical hallmarks of CDG-I deficiencies. The discovery of misregulated retina-specific proteins and the eventual loss of rod photoreceptors in the model hint at retinitis pigmentosa as a fish-specific or potentially thus far unnoticed human phenotype for ALG2-CDG.

## DISCUSSION

Studying the mechanism and consequences of protein glycosylation is highly challenging owing to the crucial importance of the pathway for protein function and, consequently, cellular and organismal development and survival. A failure of proper protein glycosylation is usually embryonically lethal, reflected by the rare occurrence of patients suffering from CDGs (Chang et al., 2018). Genetically, most CDG patients are compound heterozygous with residual functionality of the affected enzyme. The unique mutations in these key glycosylation factors carried by the patients provide essential information on the structure-function relationship of the proteins involved. To address the function and regulatory interplay of the complex glycosylation network, we have modelled a human *ALG2* mutation in medaka to establish an animal model for addressing the developmental as well as the molecular and cellular consequences of a putative hypomorphic mutation. Our homozygous alg2^p.G336*/p.G336*^ model resembles important patient phenotypes such as cranio-facial dysmorphisms, cardio-vascular problems and lethality at early juvenile stages. Mutant phenotypes were efficiently rescued by transiently providing medaka or human *alg2* mRNA at early embryonic stages.

We detected a distinct hypo-*N*-glycosylation in both the medaka mutant hatchlings and the ALG2 index patient's fibroblasts, although it was less pronounced in the patient fibroblasts. This is an interesting reflection of the molecular function of ALG2 and its mutated version, which has been established based on the patient data, in particular given the different levels of complexity (organism versus single cell type). The observed hypo-*N*-glycosylation in the medaka model resulted in two prominent molecular consequences: compensatory upregulation of the glycosylation network and a specific under-representation of proteins involved in phototransduction emanating in the specific loss of rod photoreceptors in the retina.

With respect to the compensatory effects, our proteomics results point at two routes by which the glycosylation machinery counteracted the reduced activity in the Alg2^p.G336*/p.G336*^ variant. We detected an upregulation of cytosolic proteins involved in nucleotide sugar metabolism, i.e. higher levels of the enzymes that deliver the basic glycan building blocks (Gale, Gfpt1, Alg5, Pmm2). Inside the ER lumen, thus downstream of Alg2, the machinery that processes the core *N*-glycan (Mogs, Ganab, Prkcsh) as well as members of the machinery for ER-resident protein-folding control (Hspa5, Hsp90b1) and ER-to-Golgi trafficking (Dync1i2b, Sec22b, Sec23a, Lman1, Arf5) were enriched as well (Fig. S5), apparently priming the cell for all subsequent *N*-glycan processing under these reduced glycan levels. Providing more nucleotide sugars for glycan synthesis could be a compensatory action to facilitate the synthesis of sufficient levels of the core *N*-glycan under conditions of reduced Alg2 activity. Our xCGE-LIF analysis showed reduced levels of known *N*-glycans, but the structure of the *N*-glycans transferred onto proteins was unaffected. It can thus be concluded that only properly generated, yet rare, Glc3Man9GlcNAc2 was translocated from the dolichol carrier to an Asn at the glycosylation site of nascent proteins. Despite these upregulations, the compensatory effort was not sufficient for the full maintenance of all cells and tissues in the alg2^p.G336*/p.G336*^ mutants. Our analysis revealed the framework of adjustments an organism compensating hypo-*N*-glycosylation employs, providing a glimpse into the closely intertwined regulatory topology of the glycosylation network.

Besides the upregulation of the glycosylation machinery, we detected an enrichment of the master regulator for ER stress, Hspa5 (Lee, 2005), and its interactor Hsp90b1, both of which are part of a large ER-localized multiprotein complex for protein-folding control (Meunier et al., 2002). We interpret this as a response to the higher abundance of proteins failing to pass the ER-quality checkpoint as a result of improper folding in the absence of *N*-glycosylation, as prolonged ER stress is known to induce apoptosis (Szegezdi et al., 2006).

The second prominent group of ALG2-dependent proteins relate to the progressive loss of rod photoreceptors that fail to be maintained in the alg2^p.G336*/p.G336*^ mutants. Because retinal phenotypes have been reported for several CDGs, we analysed retinal histology and cell type composition in detail and uncovered the progressive and specific loss of rod photoreceptors, a hallmark of retinitis pigmentosa (RP). RP can be caused by several different inherited mutations all resulting in a progressive loss of vision as a result of abnormalities in rod and cone photoreceptors or the retinal pigment epithelium (Parmeggiani et al., 2011). We have shown that in alg2^p.G336*/p.G336*^ mutants rod photoreceptors are initially born, but cannot be maintained. Consequently, the under-representation of retinal proteins in the mutant eyes can be interpreted as a direct consequence of the loss of cells. Alternatively, downregulated proteins could represent direct Alg2 targets that require glycosylation for rod photoreceptor survival. Strikingly, three of the prominently under-represented retinal proteins, Rhodopsin, Arrestin and a violet-sensitive opsin (Rho, Kfharr2 and Opn1sw), contain the sequon for *N*-glycosylation, but not a signal peptide by prediction (NetNGlyc 1.0; Gupta and Brunak, 2002). For Rhodopsin, however, it has already been demonstrated that its function and the survival of photoreceptors crucially depend on *N*-glycosylation of conserved asparagines at the N terminus (Asn-2 and Asn-15) (Murray et al., 2009). Targeted mutation of Asn-15 leads to defects in Rhodopsin folding and its transport to the cell surface, eventually resulting in prolonged ER stress and rod cell-specific death (Kaushal et al., 1994; Tam and Moritz, 2009). Although these points argue for a specific role of *N*-glycosylation in the maintenance of retinal function, future investigation will be required to reveal the specific molecular causes and consequences.

It is intriguing that the alg2^p.G336*/p.G336*^ variant specifically triggers apoptosis of the rod cells, whereas effects on other neuronal cell types in the eye were not apparent. This highlights that some cell types crucially depend on *N*-glycosylation of specific target proteins and, consequently, their maintenance relies on the continuous supply of sufficient levels of *N*-glycans. This is only detectable in the organismal context and underpins the high relevance of model systems for providing the full perspective when studying the multisystemic impact of glycosylation and its absence, respectively. Even though Alg2 is expressed ubiquitously, crucial targets are tissue and cell-type specific and might not be expressed at all in tissue culture models.

The complex, multisystemic phenotype of the alg2^p.G336*/p.G336*^ mutant becomes apparent only from later embryonic stages (stage 39) onwards. Its onset was efficiently rescued by injection of *alg2* mRNA at the one-cell stage. This indicates that the early production of wt Alg2 protein within the first 24 h was sufficient to rescue the late embryonic/early juvenile multisystemic phenotypes. The maternal contribution of wt Alg2 protein in homozygous mutant embryos allowed the embryo to progress through the entire early embryonic development.

Rescued alg2^p.G336*/p.G336*^ mutants showed extended viability (until they were used for histological analysis at juvenile stages 18 dph) and up to then were indistinguishable from their heterozygotic and wt siblings. Given the limited stability of injected mRNA, the rescue of embryonic phenotypes and survival up to the experimental endpoint at juvenile stages is consistent with a transient requirement of Alg2 activity during early development or a slow turnover rate of the Alg2 protein or a combination of both. ALG enzymes, and in particular ALG2, are very low abundance proteins in resting cells with a long half-life of 240 days in HeLa cells (Zecha et al., 2018). The stability of ALG2 may even be increased in complexes with ALG1 and ALG11 (Gao et al., 2004). Consequently, a transient pulse of exogenous wt *alg2* mRNA establishes a uniformly distributed, stabilized, long-lasting pool of rescuing Alg2 protein.

The patient-based medaka alg2^p.G336*/p.G336*^ mutant model described here offers a unique opportunity to investigate the linear structure-function relationships by rescue experiments with *in vitro*-modified mRNA variants. Future analyses combining mRNA-based rescue strategies with acute protein depletion will pave the way for integrating structure-function relationships with the delineation of the developmental requirement of Alg2 for establishment and maintenance of specific cell types.

## MATERIALS AND METHODS

### Animal husbandry and ethics statement

Medaka (*Oryzias latipes*) Cab strain used in this study were kept as closed stocks in accordance to Tierschutzgesetz §11, Abs. 1, Nr. 1 and with European Union animal welfare guidelines. Fish were maintained in a constant recirculating system at 28°C on a 14 h light/10 h dark cycle (Zucht- und Haltungserlaubnis AZ35-9185.64/BH).

Written informed consent was obtained for analysis of patient-derived material. This study was approved by the Ethics Committee of the Medical Faculty Heidelberg.

### Designing and cloning of sgRNAs

sgRNAs were designed with CCTop and standard conditions (Stemmer et al., 2015). The following target sites were used (PAM in brackets): *alg2 T1*, 5′-CCCGTTATTGCCGTCAACTC[TGG]; *alg2 T2*, 5′-CCGTTATTGCCGTCAACTCT[GGG]. Cloning of sgRNA templates and *in vitro* transcription was performed as detailed by Stemmer et al. (2015).

### Generation of animal lines and genotyping

One-cell-stage wt medaka (*Oryzias latipes*) Cab strain zygotes were microinjected into the cytoplasm with 150 ng/µl *Cas9* mRNA, 15 ng/µl per sgRNA, 10 ng/µl single-stranded oligodeoxynucleotide (ssODN), which introduces a premature STOP codon (underlined) upon proper integration 5′-GGTCTCTGATGAGCCTCTCCATGGCCTGGGAGAACGCCTCAGCCGTAGGCTCGCACAGGAAGCCCGTCTCCCCGTGTGCTACGCTCTCCAGAGGGCCTTAAGAGTTGACGGCAATAACGGG-3′ and 10 ng/µl *GFP* mRNA as injection tracer. GFP positive crispants were raised, outcrossed to wt and genotyped from extracted DNA of fin clip biopsies in extraction buffer [0.4 M Tris-HCl pH 8.0, 5 mM EDTA pH 8.0, 0.15 M NaCl, 0.1% SDS in distilled water with 1 mg/ml Proteinase K (Roche, 20 mg/ml)]. Genotyping PCR was performed with Q5 High-Fidelity DNA Polymerase [New England Biolabs (NEB)] with 98°C initial denaturation for 2 min, followed by 30 cycles of: 98°C denaturation for 20 s, 67°C annealing for 30 s, 72°C extension for 25 s. Primers used were: forward 5′-TCCACTTGGAGGATTGCGTC, reverse 5′-CATTTAGCTGGGGATTGGTACAC. Heterozygosity was assessed with T7 endonuclease I cleavage (New England Biolabs, M0302S) upon direct incubation of 10 µl amplicon, 2 µl 10× NEB buffer 2, 7.5 µl ddH_2_O and 0.5 µl T7E1 (NEB) for 30 min at 37°C. Fragments were size separated and analysed via gel electrophoresis. To discriminate between *alg2^+/+^*, heterozygous alg2^p.G336*/+^ and homozygous alg2^p.G336*/p.G336*^ specimens, locus amplicons (see above) were either precipitated, by adding 10% 3 M sodium acetate, 0.8× volume isopropanol, centrifuging at 18,000 ***g*** for 20 min, washing the pellet with 70% ethanol and resuspending the pellet in 1× Cutsmart buffer (NEB) overnight, or filter-column purified (AnalytikJena), and then digested with 5 Units StuI (NEB) at 37°C for 1.5 h followed by 2% agarose gel electrophoresis or verification by sequencing (MWG Eurofins Genomics).

Embryos were maintained in 1× ERM (10× ERM stock: 170 mM NaCl, 4 mM KCl, 2.7 mM CaCl_2_.2H_2_O, 6.6 mM MgSO_4_.7H_2_O, 170 mM HEPES) with 2 mg/l Methylene Blue at 26-28°C upon collection from female adults.

### Fibroblast culture maintenance

Patient and control fibroblast cells were obtained and cultivated from skin biopsies of an ALG2-CDG patient and a healthy anonymous donor, respectively. Fibroblasts were cultured in Dulbecco's modified Eagle medium (high glucose; Life Technologies) supplemented with 1% FCS (PAN Biotech) and 1% Pen/Strep under 5% CO_2_ at 37°C. Medium was replaced every 72 h.

### Alcian Blue staining

Stage 40 (9 dpf at 26°C) medaka hatchlings were anaesthetized with 1× tricaine, and the tail was clipped and processed for PCR genotyping. Specimens were fixed in 4% paraformaldehyde in PBS overnight at 4°C. Samples were dehydrated in 50% and 70% ethanol for 15 min at room temperature (RT). Cartilage was stained in 0.02% Alcian Blue 8GX (Sigma-Aldrich) in 70% ethanol and 60 mM MgCl_2_ overnight at RT. Hatchlings were washed once in 70% and 50% ethanol and eye pigments were bleached in 1% KOH, 3% H_2_O_2_ in PBS for 30 min at RT. Hatchlings were washed once with 50% ethanol and imaged under Nikon SMZ18 in 3% methylcellulose. For long-term storage, samples were dehydrated in 100% ethanol and kept at −20°C. Using Fiji (Schindelin et al., 2012), analysis of cartilage lengths (Euclidean distance) was performed according to Cline et al. (2012), i.e. individual cartilage lengths were normalized to the standard length (SL) which is the distance between the lenses of the eyes. Two-tailed nonparametric Student's *t*-test was applied for statistics.

### Imaging of vasculature anatomy

Stage 40 embryos of *zFli::GFP* (Schaafhausen et al., 2013) line and genotyped (see above) homozygous zFli::GFP;alg2^p.G336*/p.G336*^ were anesthetized in 1× tricaine and mounted laterally in glass-bottom dishes (MatTek) in 1% low melting agarose and covered in 1× ERM. Images were acquired under a Leica TCS SP8 inverted confocal laser scanning microscope at 488 nm and maximum *z*-projections of stacks were generated in Fiji.

### Haematoxylin and Eosin staining

Stage 40 (9 dpf at 26°C) medaka hatchlings were fixed in Davidson's fixative (three parts tap water, three parts ethanol, two parts formalin, one part 98% acetic acid) overnight at 4°C. Samples were dehydrated in 70%-90%-100% ethanol and xylene series. Dehydrated hatchlings were incubated in paraffin for 1 h at 60°C. All samples were embedded in paraffin blocks at RT, sectioned on a microtome at 7 µm thickness and mounted on glass slides. Following overnight incubation at 42°C, samples were stepwise rehydrated in xylene-100%-90%-70%-50% ethanol series. Slides were stained in Haematoxylin for 10 min, washed with tap water for 40 min and stained with Eosin for 2 min at RT. Samples were dehydrated with 70%-90%-100% ethanol and xylene series. Samples were preserved with Eukitt Quick-hardening mounting medium (Sigma-Aldrich). Images were taken under a DIC microscope (Leica DB5000).

### Antibody staining on cryotome sections

Samples were fixed in 4% paraformaldehyde in PTW (0.4% Tween 20 in PBS) overnight at 4°C. Samples were washed in PTW and dissected. Trunk and tails were subjected to genotyping (see above) and heads were incubated at least overnight in 30% sucrose in PTW and once in Tissue Freezing Medium (Leica, 14020108926): 30% sucrose in PTW (1:1, v/v). Samples were embedded in the same Tissue Freezing Medium and sectioned at 16 µm thickness on a cryostat (Leica CM 3050S). Slides were incubated overnight at 4°C and washed with PTW for rehydration. Slides were blocked with 10% normal goat serum (NGS) in PTW for 2 h at RT. Samples were stained with anti-Rhodopsin rabbit and mouse antibodies [1:200 (Vihtelic and Hyde, 2000) and 1:200, Millipore, MABN15], anti-Zpr1 mouse antibody (1:200, ZIRC, AB_10013803), anti-Rx2 rabbit antibody (1:200; Reinhardt et al., 2015) and anti-GS mouse antibody (1:500, Merck MAB302) in 1% NGS overnight at 4°C. Goat anti-mouse IgG (H+L) Alexa Fluor 546 (Life Technologies, A11030) and goat anti-rabbit IgG Alexa Fluor 488 (Life Technologies, A11034) or Alexa Fluor 488 goat anti-mouse IgG (H+L) (Life Technologies, A11029) and donkey anti-mouse 647 (Invitrogen, A32787) were used as secondary antibodies together with DAPI (10 µg/ml) in 1% NGS for 2 h at 37°C. Before rhodopsin antibody staining, slides were incubated in 100 mM Tris-HCl, 0.2% Tween 20 and heated up to 90°C for antigen retrieval. When combined with TUNEL staining, In Situ Cell Death Detection Kit, TMR red (Roche, 12156792910) was used according to the manufacturer's instructions. Samples were mounted in 60% glycerol in PTW and were imaged on a Leica SP8 confocal microscope. Image analysis was performed with Fiji (Schindelin et al., 2012).

### Lectin blots

#### Medaka

Total proteins were isolated from pool of 10-20 hatchlings (stage 40, anaesthetized) with RIPA Lysis and Extraction Buffer (Thermo Scientific, 89900) including cOmplete EDTA–free Protease Inhibitor Cocktail (Roche, 1183617001). Quantification was performed using the Pierce BCA Protein Assay Kit (23225). Ten micrograms of protein were loaded into each lane of 10% SDS-PAGE. Proteins were blotted on PVDF membrane (Millipore Immobilon-P, PVH00010) at 100 V for 1 h at 4°C. Membrane was blocked with 5% bovine serum albumin (BSA) in 1× TBST (TBS with 0.1% Tween 20) for 1 h at RT. As an internal control, anti-Gapdh rabbit monoclonal antibody [Cell Signaling Technology (14C10), 2118] was used at 1:1000 in blocking buffer (5% BSA in 1× TBST) for 1 h at RT. Goat anti-rabbit HRP (Agrisera, AS09602) was used at 1:5000 as a secondary antibody. After developing signal with Pierce ECL Western Blotting Substrate (Thermo Scientific, 32109), the blot was stripped in mild stripping buffer [1.5% glycine (w/v), 0.1% SDS (w/v), 1% Tween 20 (w/w), pH 2.2]. Blots were incubated in streptavidin solution (1 drop in 10 ml TBST, Vector Laboratories SP-2002) 15 min at RT to block internal biotin signal of the fish. Blots were incubated with either biotinylated concanavalin A (Con A, Vector Laboratories, B-1005) or biotinylated wheat germ agglutinin (WGA, Vector Laboratories, B-1025) at 1:1000 in TBST 2.5 h at RT. Horseradish peroxidase-streptavidin (Vector Laboratories, SA5004) was used at 1:10,000 for 30 min at RT. Signal was developed with Pierce ECL Western Blotting Substrate.

#### Fibroblast

Cell monolayers were washed with ice-cold PBS and harvested using a cell scraper. Cells were lysed for 30 min in RIPA buffer on ice and by passing the sample 20 times through a 20G needle. Samples were centrifuged for 30 min at 13,000 rpm (18,000 ***g***) at 4°C. Ten micrograms of total protein derived from patient and control fibroblasts were used for loading. Samples were mixed with 6× Laemmli buffer (375 mM Tris-HCl, pH 6.8, 6% SDS, 48% glycerol, 9% 2-mercaptoethanol, 0.03% Bromophenol Blue) and denatured at 95°C for 5 min. Extracts were analysed on a 12.5% SDS-PAGE gel and blotted onto a nitrocellulose membrane (GE Healthcare) by semi-dry electrophoretic transfer. The membrane was blocked for 1 h at room temperature with 5% milk powder in PBST (0.1% Tween 20 in PBS). After blocking, the membrane was washed and incubated with a primary antibody against β-actin (1:10,000, Sigma-Aldrich, A5441) overnight at 4°C. After washing, the membrane was incubated with secondary antibody, anti-mouse IgG conjugated with horseradish peroxidase (Santa Cruz Biotechnology, SC-516102; 1:10,000) for 45 min at RT. Protein signals were detected by light emission with Pierce enhanced chemiluminescence reagent (ECL) plus western blot analysis substrate (Thermo Fisher Scientific). After stripping the blots with 10% acetic acid for 12 min, the membrane was blocked with 5% BSA in TBST.5 (0.5% Tween 20 in TBS) for 1 h. Membranes were incubated with the biotinylated lectins Con A and WGA (Vector Laboratories) at 1:1000 in TBST.5 for 2 h at RT. The membranes were subsequently incubated with horseradish peroxidase-streptavidin for 30 min and detected with Pierce ECL plus assay kit (Thermo Fisher Scientific, 32132).

### Multiplexed capillary gel electrophoresis with laser-induced fluorescence (xCGE-LIF)

Sample preparation and quantitative analysis by xCGE-LIF was performed according to a modified version of previously described protocols (Thiesler et al., 2016; Hennig et al., 2016). Briefly, samples from pools of 20 medaka hatchlings (stage 40) and human fibroblasts (7×10^5^ cells) were lysed with RIPA Lysis and Extraction Buffer. Samples were purified with methanol/chloroform protein extraction protocol according to Wessel and Flügge (1984). Per control, mutant or patient mutation, three biological replicates were performed. The following sample preparation was carried out with the glyXprep 16 kit (glyXera, Magdeburg, Germany). *N*-glycans were released from solubilized proteins using peptide-*N*-glycosidase F and fluorescently labelled with 8-aminopyrene-1,3,6-trisulfonic acid (APTS). Excessive fluorescent dye was removed by hydrophilic interaction liquid chromatography-solid phase extraction (HILIC-SPE). The purified APTS-labelled *N*-glycans were analysed by xCGE-LIF. Data processing and normalization of migration times to an internal standard were performed with glyXtool software (glyXera, Magdeburg, Germany). *N*-glycan fingerprints (normalized electropherograms) were annotated based on migration time matching with an in-house *N*-glycan database (glyXbase) and exoglycosidase sequencing. The symbolic representations were drawn with GlycoWorkbench (Ceroni et al., 2008) according to the guideline of the Consortium for Functional Glycomics (Varki et al., 2009). To facilitate the quantitative inter-sample comparison, an aliquot of each sample was spiked in with 1 µg of a bovine asialofetuin as an internal standard prior to methanol/chloroform protein extraction. A unique and asialofetuin-derived *N*-glycan peak was used to quantitatively normalize peak intensities. The quantitative normalization was then applied to the standard *N*-glycan fingerprints using mannose-6 as a transfer peak as explained in Fig. S3.

### Full-length mRNA rescue

#### Cloning of *alg2* cDNA and mRNA synthesis

*alg2* cDNA was amplified with RT-PCR from the cDNA of wt *Oryzias latipes* Cab strain stage 18 embryos with Q5 High-Fidelity DNA Polymerase using primers extended for BamHI and XbaI recognition sequence extensions, 5′-GCCGGATCCATGGCGCGGGTGGTGTTT-3′ and 5′-GCCTCTAGATTACTGGCTGAGCATAACTACGT-3′, respectively (58°C annealing for 30 s, 70°C extension for 40 s, 35 cycles). PCR product and pCS2+ vector (Rupp et al., 1994) were digested with BamHI-HF (NEB, 20 U/ml) and XbaI (NEB, 20 U/ml) restriction enzymes and cleaned up from agarose gel with the innuPREP Gel Extraction Kit (AnalytikJena). Digested PCR product and backbone were ligated with 0.5 µl T4 DNA ligase (Thermo Scientific, 5 U/ml) in 1× ligase buffer, 10 µl end volume, for 15 min at RT. Cloned vector was transformed into Mach1-T1 cells (Thermo Fisher Scientific) via heat shock induction at 42°C for 45 s and snap-cooling on ice, 300 µl TB added and incubated for 45 min at 37°C. One hundred microlitres of the bacteria culture were plated on LB plates with ampicillin (100 μg/ml) for overnight incubation at 37°C. Individual clones were inoculated into LB medium containing ampicillin (100 µg/ml). Plasmids were extracted from bacteria culture with the QIAprep Spin Miniprep Kit (Qiagen, 27104). One clone with a successful integration was used to perform *in vitro* mRNA synthesis with mMESSAGE mMACHINE SP6 Transcription Kit (Thermo Fisher Scientific, AM1340) upon NotI-HF (NEB, 20 U/ml, R3189S) linearization and gel purification with innuPREP Gel Extraction Kit (AnalytikJena AG). The remaining plasmid was digested with 1 µl TURBO DNase (2 U/µl, Thermo Fisher Scientific) for 15 min at 37°C. RNA was cleaned up with RNeasy Mini Kit (Qiagen, 74104) and the quality of RNA was confirmed with agarose gel electrophoresis and spectrophotometry (Nanodrop).

The plasmid containing the whole cDNA of a healthy human was kindly provided by Christian Thiel (University Clinics, Heidelberg University, Germany). The RNA was synthesized *in vitro* with the mMESSAGE mMACHINE T7 Kit (Ambion, AM1344) on HpaI-linearized and gel-purified (InnuPrep, AnalytikJena) DNA as described by the manufacturers.

#### Injections into medaka

Adult heterozygous alg2^p.G336*/+^ medaka were crossed and offspring was injected at the one-cell stage with 100-200 ng/µl medaka *alg2* or 33-50 ng/µl human *ALG2* mRNA with *GFP* mRNA (10 ng/µl) as injection tracer. As injection control, offspring of the same crossing scheme was used for *GFP* mRNA injection only. Embryos were kept at 28°C. GFP negative embryos were discarded. Genotyping was performed on fin clip biopsies with Q5 High-Fidelity DNA Polymerase as stated above. The remainder of the sample was used for specific analytic procedures as detailed above.

### Mass spectrometry

#### Sample preparation and protein precipitation

Mass spectrometry (MS) was performed on whole de-yolked stage 40 hatchlings as well as stage 40 eyes. For the whole organism, stage 40 hatchlings were euthanized with tricaine, deyolked, pooled (*n*=3 biological replicates with *n*=6 each), snap-frozen in liquid nitrogen and kept at −80°C until lysis. For the measurement of eyes, both left and right eyes were dissected from 15 fish per biological replicate (*n*=4 biological replicates, 30 eyes each) and snap frozen in liquid nitrogen. Samples were lysed with 100-150 µl of RIPA Lysis and Extraction Buffer including cOmplete EDTA–free Protease Inhibitor Cocktail with the help of Qiagen TissueRaptor II. Protein lysis was incubated on ice with 50 U of benzonase nuclease (Millipore, ,E1014-5KU) for 20 min and at 37°C for 5 min. Samples were then centrifuged at 12,000 ***g*** for 10 min at 4°C. Supernatant was taken into fresh tubes and protein concentration was assessed using the Pierce BCA Protein Assay Kit. For protein precipitation, 100 µg and 30 µg of total protein for whole hatchling and eyes, respectively, were used. Samples were precipitated with a methanol/chloroform protocol according to Wessel and Flügge (1984).

#### In-solution digestion

For in-solution digestion, pellets of precipitated proteins were resuspended in 20 µl of urea buffer [8 M urea, ≥99.5%, p.a. (Carl Roth), 100 mM NaCl, ≥99.5%, p.a. (Applichem) in 50 mM triethylammonium bicarbonate (TEAB), pH 8.5 (Sigma-Aldrich)]. Cysteine thiols were subsequently reduced and alkylated by adding Tris(2-carboxyethyl)phospin (Carl Roth) to a final concentration of 10 mM and 2-chloroacetamide (≥98.0%, Sigma-Aldrich) to a final concentration of 40 mM. The solution was incubated for 30 min at RT. Sample pre-digestion was performed with lysyl endopeptidase (MS grade, Wako Chemicals), which was added in an enzyme:protein ratio of 1:40 (w/w) before the sample was incubated for 4 h at 37°C. After diluting the urea concentration to 2 M by adding 50 mM TEAB buffer, trypsin (MS grade, Thermo Fisher Scientific) was added in an enzyme:protein ratio of 1:100 (w/w) and incubated for 16 h at 37°C.

#### Dimethyl labelling

Dimethyl duplex labelling was performed bound to C18 material according to a standard protocol (Boersema et al., 2009). Briefly, digestion reaction was stopped by reducing the pH to <2 through the addition of trifluoroacetic acid (TFA, ≥99.0%, Sigma-Aldrich) to a final concentration of 0.4% (v/v) and centrifuged for 10 min at 2500 ***g*** at RT. Supernatant volume corresponding to 20 µg of total tryptic peptides per sample was loaded onto C18 StageTips (Rappsilber and Mann, 2007) containing three discs of Empore C18 material (3M). Prior to sample loading, StageTip material was successively equilibrated with 20 µl of methanol (MS grade, Carl Roth), followed by 20 µl of 50% (v/v) acetonitrile (ACN, UPLC grade, Biosolve) in 0.1% (v/v) TFA and by 20 µl of 0.1% (v/v) TFA with centrifugation for 1 min at 1500 ***g*** after each equilibration step. Loaded peptide samples were washed with 20 µl of 100 mM TEAB to shift pH for labelling and were tagged with stable-isotope dimethyl labels comprising regular formaldehyde and cyanoborohydride (28 Da shift, designated ‘light label’) or deuterated formaldehyde and regular cyanoborohydride (32 Da shift, designated ‘heavy label’) (all reagents from Sigma-Aldrich). *alg2^+/+^* samples were tagged with light labels and samples from alg2^p.G336*/p.G336*^ mutants were tagged with heavy labels, including a label swap for one of the four eye sample replicates. Labelled peptides were washed with 20 µl of 0.1% TFA and eluted from StageTip material by adding 10 µl of 50% ACN in 0.1% TFA twice with subsequent centrifugation for 1 min at 1500 ***g*** after each step. Differentially labelled samples were mixed in equal amounts, dried in a vacuum centrifuge and stored at −20°C until liquid chromatography–mass spectrometry (LC-MS) analysis.

#### LC-MS measurements

For technical reasons, whole hatchling and eye samples were analysed using slightly different LC-MS setups. Approximately 5 µg and 8.3 µg of tryptic peptides per LC-MS injection were analysed for hatchling samples (two technical replicate measurements per biological replicate with different amounts, respectively) and approximately 2 µg of tryptic peptides were analysed for eye samples. A precursor ions inclusion list was used to improve run-to-run reproducibility for all samples. All used solvents were UPLC grade.

In brief, whole hatchling samples were resuspended after vacuum centrifuge in 20% ACN/0.1% TFA and incubated for 5 min at RT, then diluted 10-fold with 0.1% TFA prior to LC-MS measurement, which was conducted using an Ultimate 3000 UPLC (Thermo Fisher Scientific) coupled to a Q-Exactive HF mass spectrometer (Thermo Fisher Scientific). Analytical LC separation was performed using an in-house packed analytical column (20 cm length, 75 μm inner diameter; Chromatographie Service) filled with 1.9 µm particle size, 120 Å pore size ReprosilPur-AQ 120 C18 material (Dr A. Maisch HPLC) and carried out for 160 min total analysis time. The chromatographic method consisted of a linear gradient of buffer B [0.1% v/v formic acid (FA), Proteochem, 10% v/v H_2_O, Biosolve in ACN] in buffer A (0.1% FA, 1% ACN in H_2_O) from 3% to 40% B in 120 min with a flow rate of 300 nl/min, followed by washing (95% buffer B) and an equilibration step. Prior to the gradient, samples were loaded to the analytical column for 20 min with 3% buffer B at 550 nl/min flow rate. Eluting peptides were analysed online using a coupled Q-Exactive-HF mass spectrometer running in DDA mode. Full scans were performed at 60,000 (m/z 200) resolution for a mass range covering 400-1600 m/z for 3e6 ions or up to a max IT of 45 ms. The full scan was followed by up to 15 MS/MS scans at 15,000 resolution with a max IT of 50 ms for up to 1e5 ions (AGC target). Precursors were isolated with a window of 1.6 m/z and fragmented with a collision energy of 27 (NCE). Unassigned and singly charged peptides were excluded from fragmentation and dynamic exclusion was set to 35 s.

For eye samples, dried peptides were resuspended in 2.5% 1,1,1,3,3,3-hexafluoro-2-propanol (Sigma-Aldrich)/0.1% TFA prior to LC-MS measurement, which was conducted using an Ultimate 3000 UPLC (Thermo Fisher Scientific) coupled to a Q-Exactive HF-X mass spectrometer (Thermo Fisher Scientific). During the LC separation, peptides were first loaded onto a trapping cartridge (Acclaim PepMap300 C18, 5 µm particle size, 300 Å pore size, Thermo Fisher Scientific) and washed for 3 min with 0.1% TFA. Analytical separation was performed using a nanoEase MZ Peptide analytical column (BEH, 20 cm length, 75 μm inner diameter, 1.7 μm particle size, 300 Å pore size, Waters) and carried out for 150 min total analysis time. The chromatographic method consisted of a linear gradient of buffer D (0.1% FA, 19.9% H_2_O, Biosolve in ACN) in buffer C (0.1% FA in H_2_O) from 5% to 38% buffer D in 132 min with a flow rate of 300 nl/min, followed by a washing (95% buffer D) and an equilibration step. Eluting peptides were analysed online by a coupled Q-Exactive-HF-X mass spectrometer running in DDA mode. Full scans were performed at 60,000 resolution for a mass range covering 350-1500 m/z for 3e6 ions or up to a max IT of 45 ms. The full scan was followed by up to 20 MS/MS scans at 15,000 resolution with a max IT of 22 ms for up to 1e5 ions (AGC target). Precursors were isolated with a window of 1.6 m/z and fragmented with a collision energy of 27 (NCE). Unassigned and singly charged peptides were excluded from fragmentation and dynamic exclusion was set to 35 s.

#### Protein identification and relative quantification with MaxQuant

Raw files were processed using MaxQuant (version 1.6.12.0; Cox and Mann, 2008) for protein identification and quantification. MS/MS spectra were searched against the Uniprot Oryzias latipes database (retrieved in February 2020, last edited in November 2019), common contaminants and an additional fasta file containing the amino acid sequence of the Usherin protein (Uniprot ID: U3R8H7) by Andromeda search engine with the following parameters: carbamidomethylation of cysteine residues as fixed modification and acetyl (protein N-term), oxidation (M) as variable modifications, trypsin/P as the proteolytic enzyme with up to two missed cleavages allowed. The maximum false discovery rate for proteins and peptides was 0.01 and a minimum peptide length of seven amino acids was required. Match between runs and requantify options were disabled. Quantification mode was with the dimethyl Lys 0 and N-term 0 as light labels and dimethyl Lys 4 and N-term 4 as heavy labels. All other parameters were default parameters of MaxQuant. Quantitative normalized ratios were calculated by MaxQuant and used for further data analysis.

#### Analysis of MS data

Perseus software (Version 1.5.6.0) was used (Tyanova et al., 2016). For the volcano plots in Fig. 4, MaxQuant normalized (total protein normalization) and log2-transformed data were filtered (at least two razor or unique peptides per protein group required) and technical replicate values were averaged, if available. One-sample *t*-test (comparison to 0, *P*-value 0.05, −1< *t*-test difference <1) was then performed and plots were produced with R and RStudio version 1.2.5042. For exclusive protein groups (only wt or only mutant), the raw intensity values for each biological replicate were normalized to the mean of unnormalized Gapdh ratios from all replicates and log2-transformed so that the proteins were plotted on the *y*-axis according to their Gapdh-normalized intensities for representation only. Exclusive hits were only considered when present in at least two biological replicates.

### Statistical analysis

No statistical methods were used to predetermine sample sizes, but our sample sizes are similar to those generally used in the field. The experimental groups were allocated randomly, and no blinding was done during allocation. Statistical analysis methods are given in the respective figure legend and main text.

## Supplementary Material

Supplementary information

Reviewer comments
