# Supplementary material for: A patient-based medaka alg2 mutant as a model for hypo-N-glycosylation
Source: Development. 2021 Jun 7;148(11):dev199385. doi: 10.1242/dev.199385 (PMC8217707; doi:10.1242/dev.199385)
Supplement: Supplementary information [file develop-148-199385-s1.pdf]

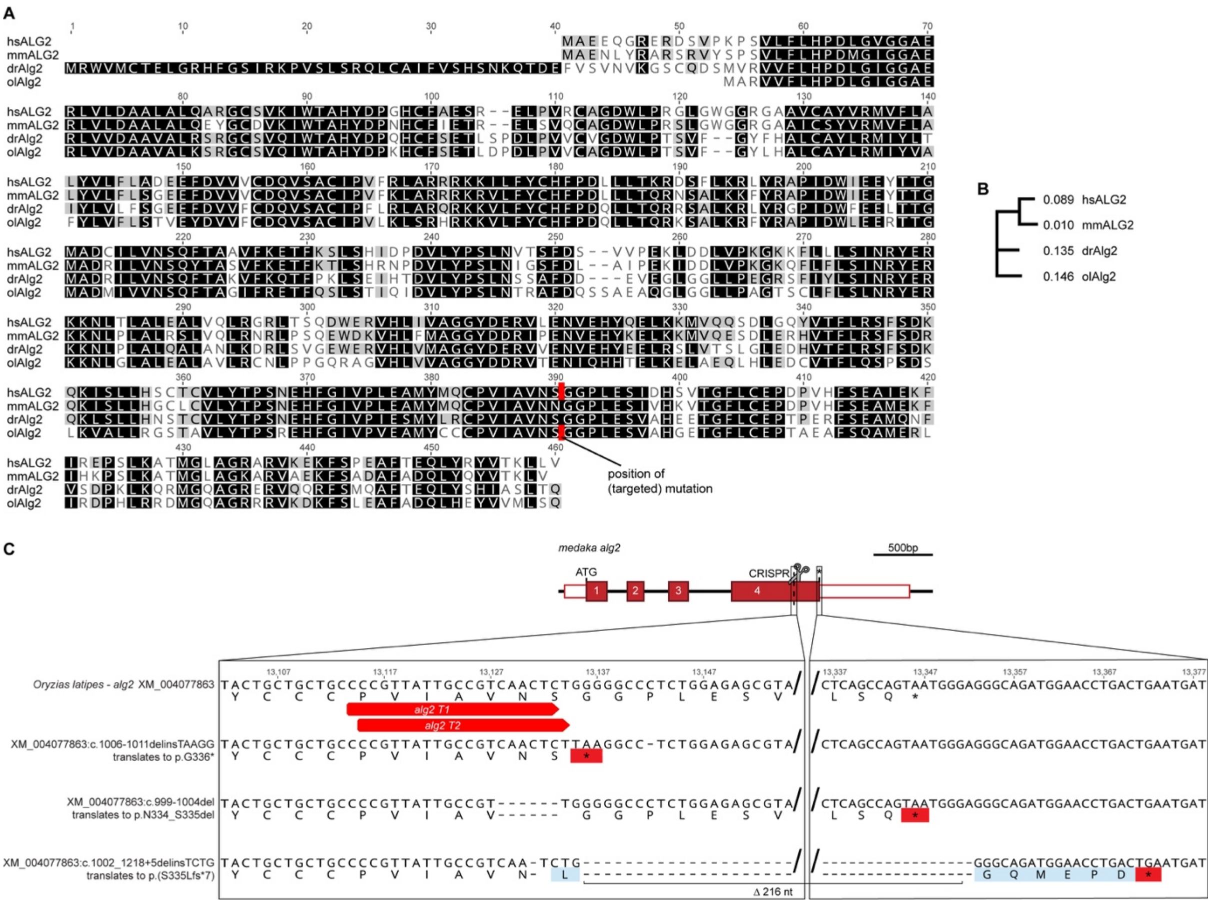

Figure S1 Evolutionary conservation and genomic sequences of *alg2* alleles.

A) The Alg2 protein is highly conserved throughout evolution as depicted by alignment of human, mouse, zebrafish and medaka Alg2 amino acid (AA) sequences. Black AA = identical, grey AA = similar. Position of human and targeted mutation (in medaka) indicated.

B) Phylogenetic tree of human, mouse, zebrafish and medaka Alg2 protein sequence.

C) Schematic representation of *Oryzias latipes alg2* locus with site of targeted mutagenesis indicated (grey scissors; red box, coding exons; white box, UTR). Zoom into the genomic sequence at the site of mutagenesis in wild-type and stable alleles of the three mutant *alg2* alleles. Potential aminoacid sequence given below codons of open reading frame. red, premature STOP, light blue, amino acids resulting from frameshift. Nomenclature according to (Dunnen *et al*, 2016).

Development • Supplementary information

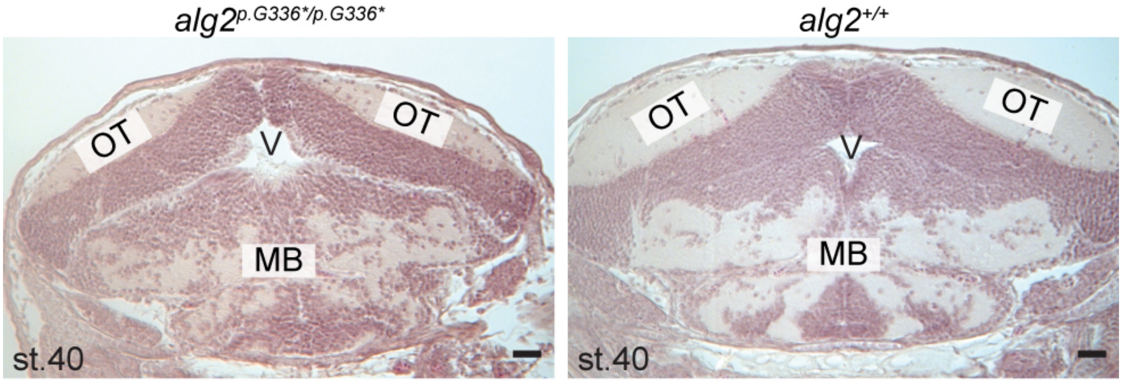

Figure S2 ***Alg2$^{p.G336*/p.G336*}$*** **homozygotes show reduced white matter.** Hematoxylin and eosin staining on transverse sections of *alg2$^{p.G336*/p.G336*}$* (left) and wild-type (wt) embryos (right) at stage 40. Note overall reduction of white matter most pronounced in the optic tectum (OT). Larger ventricle (V) and not well confined lining as compared to wt. Scale bar = 50 µm

Development • Supplementary information

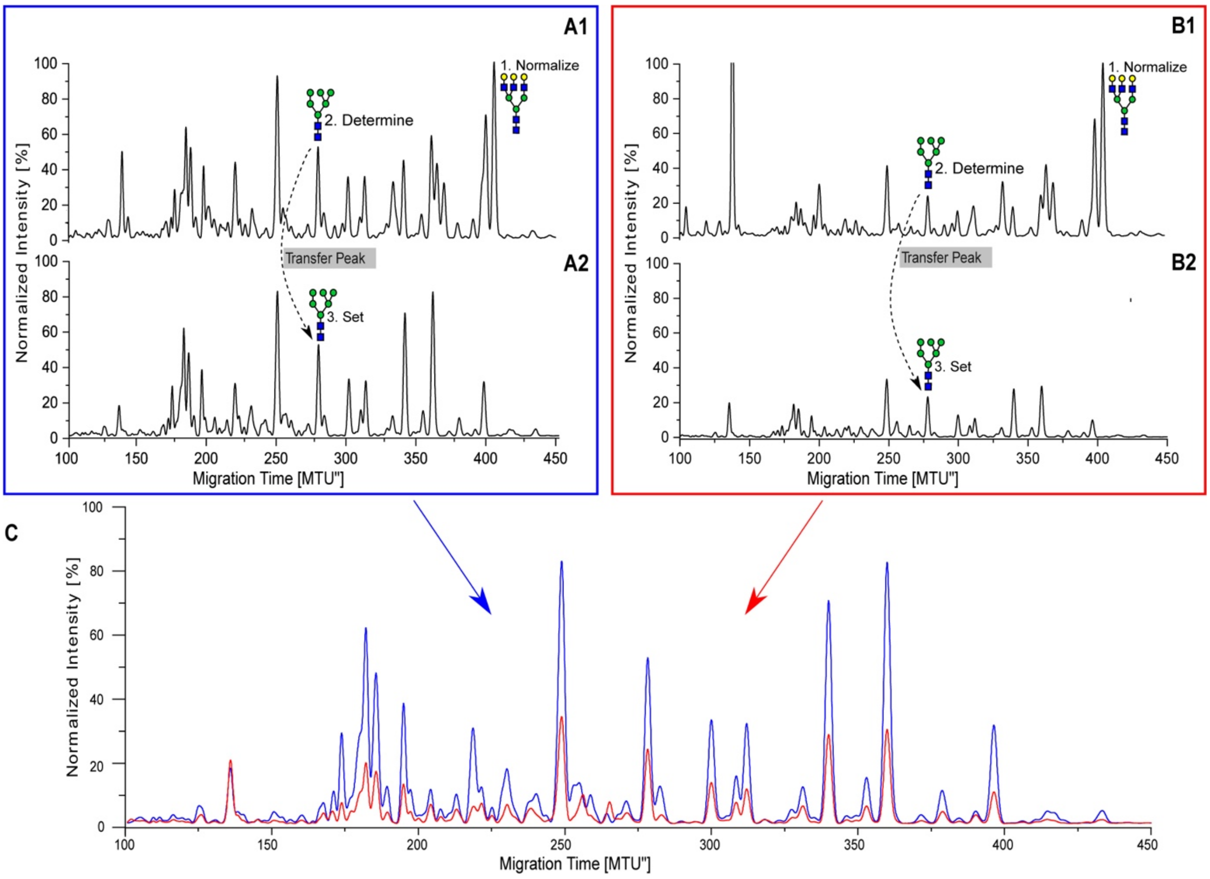

Figure S3 Normalization of xCGE-LIF electropherogram.

Schematic overview of the quantitative normalization for *N*-glycan fingerprints using an internal standard. A1/B1: *N*-glycan fingerprints of sample A and B spiked with internal standard (bovine asialofetuin). Intensities are normalized to the asialofetuin-derived *N*-glycan peak (A3G3). A2/B2: A sample-derived *N*-glycan peak (Man6) is used to transfer the quantitative normalization to *N*-glycan fingerprints not containing the internal standard. C: Overlay of samples (A2 and B2) comprising a quantitative normalization enabling inter-sample comparison.

Development • Supplementary information

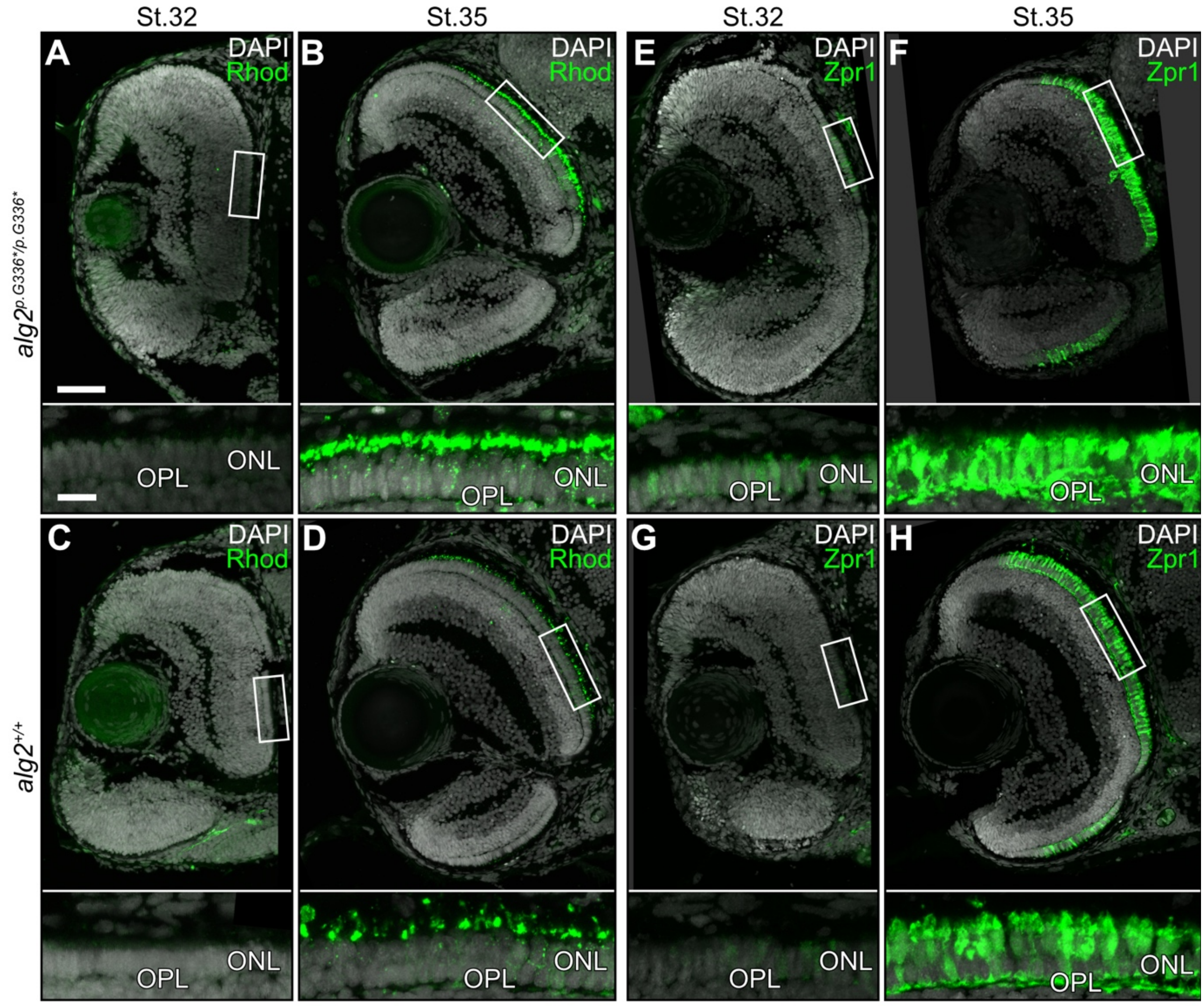

Figure S4 Separation of rod and cone photoreceptors is affected in *alg2*$^{p.G336*/p.G336*}$ mutant retinae.

Time series of retinal development as depicted by DAPI and immunohistochemistry staining against rod (Rhodopsin) and cone (Zpr1) specific markers on cryotome sections in homozygous *alg2*$^{p.G336*/p.G336*}$ (A-B, E-F) and wildtype *alg2*$^{+/+}$ (C-D, G-H) siblings at stage 32 (A, E, C, G) and stage 35 (B, F, D, H).

The onset of separation of rod and cone photoreceptors at stage 35 (D, H) is failing in *alg2*$^{p.G336*/p.G336*}$ mutant embryos (B, F). Rhod and Zpr1 staining are irregular in *alg2*$^{p.G336*/p.G336*}$ mutant embryos at stage 35 due to affected ONL lamination.

ONL, outer nuclear layer; OPL, outer plexiform layer; boxes depict area of zoom; scale bars = 50 μm and 10 μm in zoomed panels.

Development • Supplementary information

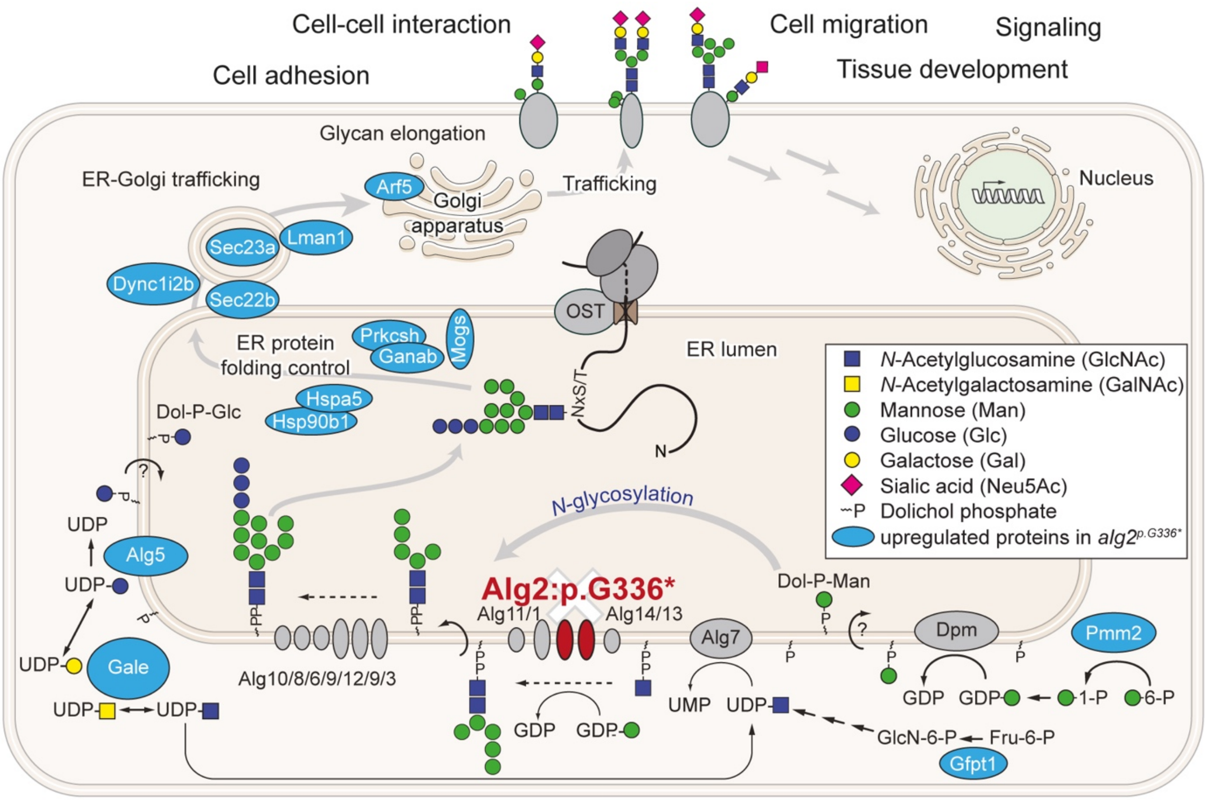

Figure S5 The upregulation of the glycosylation machinery as compensatory effort in the *alg2*$^{p.G336*/p.G336*}$ mutants.

Schematic overview of the altered *N*-glycosylation machinery detected in *alg2*$^{p.G336*/p.G336*}$ mutants with the upregulated proteins detected in the proteomics analysis (light blue) put into context. Two main routes affecting glycosylation can be seen: upregulation of basal enzymes allocating nucleotide sugars (Gale, Gfpt1, Alg5 and Pmm2) and following the translocation of the oligosaccharide from the lipid-linked donor to the Asn of a nascent protein, factors relevant for core *N*-glycan processing (Mogs, Ganab, Prkcsh), ER resident protein folding control (Hspa5, Hsp90b1) and ER-to-Golgi trafficking (Sec22b, Dync1i2b, Sec23a, Lman1, Arf5) were highly abundant in the *alg2*$^{p.G336*/p.G336*}$ mutants.

OST, Oligosaccharyltransferase

Table S1 Full length edaka and human alg2 mRNA rescue homozygous *alg2*$^{p.G336*/p.G336*}$ survival.

injection	-	-	*olAlg2* mRNA		*hsAlg2* mRNA	
			100 ng/µl	200 ng/µl	33 ng/µl	50 ng/µl
total n collected/injected	40	40	37	59	39	32
total dead (until 4 dph)	14	15	9	17	7	3
total genotyped at 4dph	26	25	28	42	32	29
wt *alg2*$^{+/+}$	10	10	7	14	11	8
heterozygous *alg2*$^{p.G336*/+}$	16	15	16	16	15	18
homozygous *alg2*$^{p.G336*/p.G336*}$	0	0	5	12	6	3

Offspring derived from *alg2*$^{p.G336*/+}$ incrosses individually genotyped at 4 days post hatching (dph). No homozygous *alg2*$^{p.G336*/p.G336*}$ could be detected in the uninjected control batches. Exogenous supply of medaka and human full length *alg2* mRNA successfully rescued survival close to Mendelian distribution.

Table S2

N-glycan structures identified in lysates from medaka wild-type and *alg2*$^{p.G336*/p.G336*}$ hatchlings (biological triplicates). Relative intensities are based on normalization to internal standard. Symbolic representation of *N*-glycan structures were drawn with GlycoWorkbench Version 1.1, following the guideline of the Consortium for Functional Glycomics (Varki *et al*, 2009). Glycan names are adapted from the Oxford nomenclature.

Click here to download Table S2

Table S3

Proteomics results of stage 40 whole hatchlings: differential proteomics *alg2*$^{p.G336*/p.G336*}$/wt *alg2* $^{+/+}$ (sheet 1) and exclusive hits (sheet 2). Enucleated eye samples: differential proteomics *alg2*$^{p.G336*/p.G336*}$/wt *alg2*$^{+/+}$ (sheet 3) and exclusive hits (sheet 4).

Click here to download Table S3

Development • Supplementary information
