# Supplementary material for: A patient-based medaka alg2 mutant as a model for hypo-N-glycosylation
Source: Development. 2021 Jun 7;148(11):dev199385. doi: 10.1242/dev.199385 (PMC8217707; doi:10.1242/dev.199385)
Supplement: Reviewer comments [file develop-148-199385_review_history.pdf]

Development

A patient-based medaka *alg2* mutant as a model for hypo-*N*-glycosylation

Sevinç Gücüm, Roman Sakson, Marcus Hoffmann, Valerian Grothe, Clara Becker, Kaisa Pakari, Lars Beedgen, Christian Thiel, Erdmann Rapp, Thomas Ruppert, Thomas Thumberger and Joachim Wittbrodt

DOI: 10.1242/dev.199385

Editor: Stephen Wilson

Review timeline

Original submission:	24 December 2020
Editorial decision:	3 February 2021
First revision received:	8 April 2021
Accepted:	4 May 2021

Original submission

First decision letter

MS ID#: DEVELOP/2020/199385

MS TITLE: A humanized medaka alg2 mutant as novel model for hypo-N-glycosylation

AUTHORS: Sevinç Gücüm, Roman Sakson, Marcus Hoffmann, Valerian Grothe, Lars Beedgen, Christian Thiel, Erdmann Rapp, Thomas Ruppert, Thomas Thumberger, and Joachim Wittbrodt

Best wishes for the new, and hopefully better, new year.

I have now received all the referees' reports on the above manuscript, and have reached a decision. The referees' comments are appended below, or you can access them online: please go to BenchPress and click on the 'Manuscripts with Decisions' queue in the Author Area.

As you will see, the referees express considerable interest in your work, but have some significant criticisms and suggestions for improvements to your manuscript. If you are able to revise the manuscript along the lines suggested, I will be happy receive a revised version of the manuscript. Please also note that Development will normally permit only one round of major revision.

We are aware that you may be experiencing disruption to the normal running of your lab that make experimental revisions challenging. If it would be helpful, we encourage you to contact us to discuss your revision in greater detail. Please send us a point-by-point response indicating where you are able to address concerns raised (either experimentally or by changes to the text) and where you will not be able to do so within the normal timeframe of a revision. We will then provide further guidance. Please also note that we are happy to extend revision timeframes as necessary.

Please attend to all of the reviewers' comments and ensure that you clearly highlight all changes made in the revised manuscript. Please avoid using 'Tracked changes' in Word files as these are lost in PDF conversion. I should be grateful if you would also provide a point-by-point response detailing how you have dealt with the points raised by the reviewers in the 'Response to Reviewers' box. If you do not agree with any of their criticisms or suggestions please explain clearly why this is so.

© 2021. Published by The Company of Biologists under the terms of the Creative Commons Attribution License (https://creativecommons.org/licenses/by/4.0/).

Reviewer 1

Advance summary and potential significance to field

In manuscript DEVELOP/2020/199385 by Gücüm et al, the authors have modelled a congenital disorder of glycosylation called ALG2-CDG in the teleost medaka (Oryzias latipes). They first utilized CRISPR/Cas9 genome editing to generate a hypomorphic mutation from an index patient into the α-1,3/1,6-mannosyltransferase (ALG2) gene in medaka and analyzed its developmental consequences. Careful phenotypic characterization of this medaka Alg2 hypomorphic allele revealed the onset of multisystemic defects prior to hatching at stage 39 and showed some overlap with the phenotypes observed in human patients. Using lectin-binding assays, the authors showed hypo-N-glycosylation in Alg2 mutant fish but not in fibroblasts from an ALG2-CDG patient. A detailed analysis of N-glycans structures using xCGE-LIF approach provided a more nuanced picture: In the medaka model, the Alg2 mutation resulted in a pronounced hypo-N-glycosylation affecting all N-glycan structures to a comparable degree. The authors conclude from this dataset that patient cells showed a similar effect on N-glycans although with less severity compared to the fish model (please see below a potential concern with this interpretation). Proteomic analysis on whole hatching and enucleated eye samples showed over-representation of some proteins involves in N-glycosylation machinery and under-representation of a number of proteins involved in phototransduction and implicated in retinitis pigmentosa. Using H&E and immunofluorescent staining combined with TUNEL assay, they found a loss of rod photoreceptors due to apoptosis in Alg2 mutants. Finally, they showed that the Alg2 mutant phenotypes can be rescued by exogenous Alg2 mRNA from medaka or human.

ALG2 is a highly conserved enzyme required for the Lipid-linked Oligosaccharide synthesis during N-glycosylation. It mannosylates Man1GlcNAc2-dolichol diphosphate and Man2GlcNAc2-dolichol diphosphate to form Man3GlcNAc2-dolichol diphosphate. Its loss-of-function causes a congenital disorder of N-glycosylation (ALG2-CDGIi) which is characterized by iris coloboma, developmental delay and coagulopathy. Considering the scarcity of available models to explore the ALG2 loss-of-function defects and lack of an FDA-approved therapy, the current work provides a nice model that recapitulates some of the human disease phenotypes and provides a platform for mechanistic and preclinical studies. Moreover, the sophisticated glycomic analysis combined with the proteomic data provide novel information on the compensatory increase in some proteins in the N-glycosylation machinery, which might be relevant not only to this type of CDG but to others as well. The data are generally of high quality and are presented in a visually appealing manner. Moreover, the Discussion is well written (for the most part). Besides these strengths, there are some concerns about the manuscript, including issues related to data interpretation, which the authors should consider addressing.

Comments for the author

Major points

1. Figure 3C-F: The description provided for human fibroblasts does not seem to reflect the data accurately. First, unlike medaka, the mutant human fibroblasts show increase in some N-glycan glycoforms compared to control fibroblasts (5, 7, 15, 16) and do not show a change in the abundance of some others (14, 19, 37). Moreover, calling 0.5% "marginal change" implies that this is a significant/meaningful change. However, this is likely to be within the normal range. In line with the data shown in Figure 3B, an alternative description and interpretation of the data might be that since some forms of N-glycan are increased and others decreased, the overall abundance of complex N-glycans is not changed in patient fibroblasts. For high mannose N-glycans, the balance is tilted towards an overall decrease. The authors should consider modifying the description of Figure 3D-F. Given the data, one wonders whether we can use hypo-N-glycosylation for the patient fibroblasts, given the data. It's certainly not "severe hypo-N-glycosylation" for these cells, as mentioned on line 280. These issues potentially affect the title and the abstract as well, because although hypo-N-glycosylation is clearly seen in the medaka model, in human fibroblasts only some forms of N-glycans are reduced and others are increased. I suggest that the authors go through the text carefully to make sure this issue is addressed throughout the manuscript.

2. The authors concluded that rod photoreceptors were initially formed but were eliminated by apoptosis at a later stage. To reach this conclusion, the authors will need to show the presence

© 2021. Published by The Company of Biologists under the terms of the Creative Commons Attribution License (https://creativecommons.org/licenses/by/4.0/).

of specific markers for rod photoreceptors at an earlier stage. Otherwise, based on the absence of a marker (Rhod) and just the location of the cells one cannot conclude that rod photoreceptors were born.

3. Lines 393-395, Figure 5B: The authors mention that cone cells were not affected. However, based on the images, it looks like Zpr1 is stronger in WT along the ONL and shows a somewhat different pattern compared to the mutant eye. Therefore, the conclusion is not supported by these data. The authors should either revised the text to acknowledge potential defects in cone cells or provide additional data and quantifications to support the notion that cone cells are not affected.

4. Figure 6A: The GS staining looks quite different in Alg2 mutant as compared to WT throughout the section. This is not reflected in the description provided in the figure legend and in the authors' conclusion from this figure.

Other points
1. Title: Is humanized the appropriate term here? One usually sees this adjective when a gene or a specific cell type in an organism is replaced by the corresponding gene or cell type from humans (like a humanized monoclonal antibody or pigs with a humanized liver). I'm not sure whether this is the correct word for a knock-in allele of a gene in a model organism.

2. Line 45: photoreceptors “,” resulting “in” retinitis pigmentosa …

3. Line 71: When glycosylation is affected in patients, “they” suffer from Congenital …

4. Line 78: “A number of” key steps … (“Few” has a negative connotation here).

5. Line 85: The preferred term for the switch in the location of LLOs is “flipping”. “Internalization” would suggest something like endocytosis, but here the LLO does not lose its link to the ER membrane; only what is outside is flipped to the inside.

6. Line 90: to form “hybrid and” complex glycan …

7. Lines 124-126: I suggest that the authors rephrase “at the basis” and the sentence after “Striking”. The messages are not clear (especially for the sentence after Striking).

8. Line 130: I'm not sure why “initially” is used here.

9. Line 135-136: The first sentence of this paragraph is too broad and not accurate. There are many examples of glycosylation related proteins that are not embryonic lethal even when null alleles are used. Perhaps the authors should add “many” before “glycosylation related proteins” in this sentence.

10. Figure 1F: Does each square/circle represent one animal? Please mention in the legend.

11. Line 197: Are homozygous mutants 100% lethal? How many animals have been used for each of three mutants generated in this study to reach this conclusion?

12. Line 237: “Fig. S2” instead of Fig. 32.

13. Lines 383-283, Figure S3: How many animals have the authors analyzed to reach the conclusion that white matter is reduced? Was the phenotype 100% penetrant? It's better to mention the animal numbers for staining data throughout the manuscript.

14. Lines 508-510: Alternatively, the retinitis pigmentosa phenotype might be specific to fish.

15. Line 522: Perhaps “as well as” should be used instead of “as much as”.

16. Line 524: “all human phenotypes” seems to be somewhat exaggerated. Perhaps the authors can list the specific phenotypes that are recapitulated in their model.

© 2021. Published by The Company of Biologists under the terms of the Creative Commons Attribution License (https://creativecommons.org/licenses/by/4.0/).

17. Lines 569-571: Listing cytoplasmic proteins like Arrestin here can be misleading. The sequon for N-glycosylation is likely to be only meaningful for proteins that go through the ER. It's also better to end this paragraph with a concluding sentence.

Reviewer 2

Advance summary and potential significance to field

This is a well-executed study which model the ALG2-CDG in fish by introducing a mutation at the same position in the alg2 gene as an human index patient.
Homozygous mutants displayed abnormal facial skeleton, cardiovascular system rod photoreceptors - and the resulting phenotype recapitulates ALG2-CDG patients.
Proteome analysis indicated compensatory expression of glycosylation enzymes. Rod cells in the retina and altered abundance of glycoproteins directly implicated with photoreceptor function was observed. This study makes a good case to use this line as a disease model for ALG2-CDG and retinitis pigmentosa. Since the effects can be effectively rescued by mRNA injection it will also serve as an efficient tool to test the activity of modified mRNA variants.

Comments for the author

Minor points:
1. I lack a discussion on the effects on the truncated protein; what active sites are missing etc.
2. One can assume that ALG2 is ubiquitously expressed in Medaka as well as other vertebrates – but is this investigated in the fish model (eg Medaka or Zebrafish)? Or is this assumption built solely on interference from mammalian studies?
3. Mutant embryos injected with wt mRNA survive at least to day 18dph (row 475). Assuming that the phenotype is largely normal - the larva swimming and eating – does this result suggest that ALG2 might be dispensable for adult life in Medaka? Was no mRNA injected mutant ever followed to adult stages?

Reviewer 3

Advance summary and potential significance to field

Manuscript by Gücüm, et al., "A humanized medaka alg2 mutant as novel model for hypo-N-glycosylation" addresses the role of ALG2 mannosyl transferase in medaka animal model during development using CRISPR edited genetic knock in lines and a range of biochemical and developmental genetic approaches. In addition proteomics and glycan analysis in patient fibroblasts was performed. The study is of general interest to clinicians and developmental biologists and will expand knowledge on the role of ALG2.

Comments for the author

The manuscript is neatly written and data are nicely presented and analyzed. However, there are numerous points that need to be address to clarify results and align claims with presented data.

Major points:
1. The model: The study presents generation of a new medaka fish model for a congenital disorder of glycosylation (CDG-Ii) by ssODN CRISPR editing to introduce a stop codon in medaka alg2 gene in an equivalent position to human ALG2 that was observed in a patient. Several alleles are induced and one is analyzed. This is very nicely done, however some clarifications throughout the manuscript are needed. First it is not generally accepted to refer to this type of a model as "humanized medaka model". To be accurate the authors would need to generate a medaka model where the endogenous alg2 locus is replaced by the human ALG2 gene that carry the patient's variant. Alternatively, the authors could change the title.
Second, it is not clear from the results section why this (ssODN) approach was used.

© 2021. Published by The Company of Biologists under the terms of the Creative Commons Attribution License (https://creativecommons.org/licenses/by/4.0/).

2. It is confusing throughout the manuscript what the authors have concluded about the nature of the model.
They refer to it as viable but homozygotes die at early larval stages. It cannot be both ways. There is also no information on the patient variant (beyond the reference) and its impact on protein presence. Is it a hypomorph or a hyploinsuficiency? Is there any evidence to substantiate it or it is unknown. My own literature search showed that this model is not faithfully recapitulating the patient situation because the patient is a trans-heterozygote for a variant leading to a stop codon and paternal variant that appears to be unstable and not contributing enzymatic activity. All these are very important nuances that could shed light on the ALG2 function and need to be addressed and discussed in the manuscript. In this context medaka model would be only testing the homozygote recessive version of one of the patient alleles.

3. The characterization of the phenotypes: The claims are overreaching, e.g. “fully resemble the multisystemic phenotype” (120) while we later learn that only a few phenotypes were tested and quite superficially described.
This needs to be addressed throughout the text. Specifically, one of the core components of the CDG-Ii (and other CDGs) is myasthenic syndrome. This is not discussed or analyzed in any way. It would be extremely valuable to evaluate muscle markers and structure in this model. Do older hatchlings move in a normal way?

4. The eye phenotype is very interesting and has not been associated with ALG2 clinical symptoms, although CDGs commonly affect vision in affected persons.
The authors state in the discussion that they “have shown that in alg2hypo mutants rod photoreceptors are initially born, but not maintained”. This is not fully supported by the data presented. Time course imaging of retinal IHC would be needed to show the initial presence of rod photoreceptors, followed by progressive loss over time. The study would benefit from time course imaging and quantitative assessment.

5. The rescue: This is a very important part of model validation and it clearly shows that WT medaka mRNA can reverse some aspects of the overall gross morphology phenotype. The human ALG2 rescue with n=2 is substandard. Appropriate power analysis needs to be conducted and adequate number of animals analyzed.
Alternatively, this can be removed along with the claims accompanying it (e.g. 525). How similar are the human and medaka proteins? Does a patient mRNA variant rescue the medaka phenotype? This would be a very important experiment especially if evaluated by biochemical or cell-level readouts.

6. Glygo- and proteomics analyses: Very elegant study of glycosylation and proteomics is presented. What is the ALG2 protein level in patient fibroblasts and alg2 in medaka samples? This would be important to discuss in the main manuscript.

Minor points:
1. (178) How is the SL defined? Methods section or legend would benefit from description.
2. (189) “enlarged liver” is not defined by any marker analysis or quantification.
3. (197) Is there evidence that medaka hatchlings need blood circulation at this stage to survive?
Reference?
4. (207) Needs language editing “supposed to suffer”, Also, (33), (358), and others.
5. (280) The use of “hypo-N-glycosylation” is very effective, but I cannot understand why the authors use proxy for homozygote mutant. Very confusing and needs to be revised.
6. (302) Cartilage typically is made of type II collagen and others, but type I is only marginally involved.
Needs correction.
7. (303) Reference typo 8. (328) Sec22b and Sec23a are both trafficking machinery components and are cytosolic proteins when inactive. Sec23a is clearly not processing proteins in ER. Revise here and in all other places.
9. (401) The WT is not matching the plane of section to mutant, so the two images are not comparable.
10. (408) The layers appear to be about 30% thinner, this is more than just “slightly” thinner. Quantification of sufficient number of animals would be needed.

© 2021. Published by The Company of Biologists under the terms of the Creative Commons Attribution License (https://creativecommons.org/licenses/by/4.0/).

11. (423) What is the nature of Rx2? Some explanation would help.
12. (431) It is very hard to see anything in these images. Using gray scale in individual staining panels and color in merged would help with assessment of data. In B I see a clear difference in packing of cell, tight pack in WT, diffuse but patterned retina in mutant. Number of layers are distended as seen in DAPI. What does it mean? Conclusions should be saved for discussion and not figure legends. Here, I would rather see a brief explanation of tools or methods to help me evaluate the data.
13. (465) How much mRNA was actually injected to each embryo?
14. (487) Overexpression of Alg2 in WT – how was it evaluated? What were the readouts?
15. (592) Revised “sacrificed” (religious or cult term) to “euthanized” or “reach experimental end point”
16. How were the lectin-blotting experiments conducted and quantified?
17. Figure 3E-F: the graphs axes scale don’t match up with the text. It’s unclear whether there was some sort of normalization.
18. This is very confusing: “we accordingly use alg2hypo as a proxy for the medaka homozygous mutant” Is the homozygous mutant a hypomorph? Or are the heterozygous mutants used as the hypomorph?
19. Are there any phenotypes in the heterozygous mutants?
20. Figure 6: It is unclear from the legend what glutamine synthase is labeling. It is difficult to see the bilayer of cells referred to in the text.

First revision

Author response to reviewers' comments

Reviewer 1

Advance Summary and Potential Significance to Field:
In manuscript DEVELOP/2020/199385 by Gücüm et al, the authors have modelled a congenital disorder of glycosylation called ALG2-CDG in the teleost medaka (Oryzias latipes). They first utilized CRISPR/Cas9 genome editing to generate a hypomorphic mutation from an index patient into the α-1,3/1,6-mannosyltransferase (ALG2) gene in medaka and analyzed its developmental consequences. Careful phenotypic characterization of this medaka Alg2 hypomorphic allele revealed the onset of multisystemic defects prior to hatching at stage 39 and showed some overlap with the phenotypes observed in human patients. Using lectin-binding assays, the authors showed hypo-N-glycosylation in Alg2 mutant fish but not in fibroblasts from an ALG2- CDG patient. A detailed analysis of N-glycans structures using xCGE-LIF approach provided a more nuanced picture: In the medaka model, the Alg2 mutation resulted in a pronounced hypo-N-glycosylation affecting all N-glycan structures to a comparable degree. The authors conclude from this dataset that patient cells showed a similar effect on N-glycans although with less severity compared to the fish model (please see below a potential concern with this interpretation). Proteomic analysis on whole hatching and enucleated eye samples showed over-representation of some proteins involves in N-glycosylation machinery and under-representation of a number of proteins involved in phototransduction and implicated in retinitis pigmentosa. Using H&E and immunofluorescent staining combined with TUNEL assay, they found a loss of rod photoreceptors due to apoptosis in Alg2 mutants. Finally, they showed that the Alg2 mutant phenotypes can be rescued by exogenous Alg2 mRNA from medaka or human.

ALG2 is a highly conserved enzyme required for the Lipid-linked Oligosaccharide synthesis during N-glycosylation. It mannosylates Man1GlcNAc2-dolichol diphosphate and Man2GlcNAc2-dolichol diphosphate to form Man3GlcNAc2-dolichol diphosphate. Its loss-of-function causes a congenital disorder of N-glycosylation (ALG2-CDGIi) which is characterized by iris coloboma, developmental delay and coagulopathy. Considering the scarcity of available models to explore the ALG2 loss- of-function defects and lack of an FDA-approved therapy, the current work provides a nice model that recapitulates some of the human disease phenotypes and provides a platform for mechanistic and preclinical studies. Moreover, the sophisticated glycomic analysis combined with the proteomic

© 2021. Published by The Company of Biologists under the terms of the Creative Commons Attribution License (https://creativecommons.org/licenses/by/4.0/).

data provide novel information on the compensatory increase in some proteins in the N-glycosylation machinery, which might be relevant not only to this type of CDG but to others as well. The data are generally of high quality and are presented in a visually appealing manner. Moreover, the Discussion is well written (for the most part). Besides these strengths, there are some concerns about the manuscript, including issues related to data interpretation, which the authors should consider addressing.

Reviewer 1 Comments for the Author:
Major points
1. Figure 3C-F: The description provided for human fibroblasts does not seem to reflect the data accurately. First, unlike medaka, the mutant human fibroblasts show increase in some N-glycan glycoforms compared to control fibroblasts (5, 7, 15, 16) and do not show a change in the abundance of some others (14, 19, 37).
Moreover, calling 0.5% "marginal change" implies that this is a significant/meaningful change. However, this is likely to be within the normal range. In line with the data shown in Figure 3B, an alternative description and interpretation of the data might be that since some forms of N-glycan are increased and others decreased, the overall abundance of complex N-glycans is not changed in patient fibroblasts.
While we agree that there is no significant change in complex-type N-glycosylation of the human fibroblasts, the 53 % reduction of high-mannose-type N-glycans still results in an overall decrease of N-glycosylation of 19 %, which can be considered as a significant hypo-N-glycosylation.
We agree with the referee that the text describing the N-glycan fingerprints (shown in figure 3 C, D) was potentially misleading since it was describing the relative composition (e.g., percentage of high-mannose-type N-glycans in one sample).
These numbers were only indirectly accessible from the figures. Therefore, we extended the experimental basis and extensively revised the figure and its description in the revised version of the manuscript. The revised paragraph now coherently reports the absolute changes observed by comparison of control and mutant samples. Also, we extended the initial dataset and now provide the results of biological triplicates.

For high mannose N-glycans, the balance is tilted towards an overall decrease. The authors should consider modifying the description of Figure 3D-F. Given the data, one wonders whether we can use hypo-N-glycosylation for the patient fibroblasts, given the data. It's certainly not "severe hypo-N-glycosylation" for these cells, as mentioned on line 280.
We thank the referee for this comment and have modified the revised manuscript accordingly. We present the strong hypo-N-glycosylation in the medaka model the manuscript is describing and a 19% reduction of overall N-glycosylation in human patient derived fibroblasts that, taken together, show a prominent hypo-N- glycosylation in both contexts. We clearly mention the difference and refer to the differences in complexity of the models employed.

These issues potentially affect the title and the abstract as well, because although hypo-N-glycosylation is clearly seen in the medaka model, in human fibroblasts only some forms of N-glycans are reduced and others are increased. I suggest that the authors go through the text carefully to make sure this issue is addressed throughout the manuscript.
In the revised version of the mansucript we have taken care to precisely deal with this point raised by the referee. We have made sure that this issue is addressed properly throughout the revised manuscript. For the molecular comparison to the human situation, patient fibroblasts are the only point of reference. In the absence of a human model of comparable cellular complexity, the medaka model can consequently be only be compared to fibroblasts. Strikingly, however, we detected a consistent pattern in the reduction of high-mannose-type N-glycans, irrespective of the cellular complexity.

2. The authors concluded that rod photoreceptors were initially formed but were eliminated by apoptosis at a later stage. To reach this conclusion, the authors will need to show the presence of specific markers for rod photoreceptors at an earlier stage. Otherwise, based on the absence of a marker (Rhod) and just the location of the cells one cannot conclude that rod photoreceptors were born.
We thank the referee for this comment and have extended our analysis accordingly. We have included new display items: figure 5, figure 7 and supplemental figure S4 to extensively address the point raised by this referee. We now show the time course of retinal/photoreceptor

© 2021. Published by The Company of Biologists under the terms of the Creative Commons Attribution License (https://creativecommons.org/licenses/by/4.0/).

differentiation in figure 5 and supplemental figure S4 comparing the homozygous mutants to wild type siblings. We use DAPI nuclear staining to highlight retinal lamination complemented by staining for rod photoreceptors (Rhodopsin) and cone photoreceptors (Zpr1). Rod photoreceptors are initially born and subsequently reduced and eventually lost in the central retina, while cone photorecpetors remain uaffected. Already at the onset of cone differentiation we observe an apparent failure of the mutant photoreceptor layer (compare figure 5B/F) to segregate the respective nuclei into an inner rod and outer cone layer. We have extensively revised the corresponding paragraph in the revised version of the manuscript to highlight the failure to eventually maintain initially born rod photoreceptors. In the new figure 7 we correlate the disappearance of rod photoreceptors to the progressive detection of apoptotic cells after the onset of the expression of rod specific markers. This further substantiates the statement of the initial birth of rod photoreceptors that fail to be maintained subsequently.

3. Lines 393-395, Figure 5B: The authors mention that cone cells were not affected. However, based on the images, it looks like Zpr1 is stronger in WT along the ONL and shows a somewhat different pattern compared to the mutant eye. Therefore, the conclusion is not supported by these data. The authors should either revised the text to acknowledge potential defects in cone cells or provide additional data and quantifications to support the notion that cone cells are not affected.
We thank the referee for pointing this out. In the all new figure 5 we have taken care to present the respective stainings over time. While a difference in the Zpr1 staining comparing mutant and wildtype is apparent, our analysis highlights that this is due to dying rod photoreceptors (pyknotic nuclei, highlighted by white arrowheads in figure 5D). While regularly spaced rod photoreceptors in the wild type retina direct the regular spacing of cones, the successive loss of rod photoreceptors in the mutant consequently affects on the spacing of the cones. We now clearly state that in the corresponding sections of the text and figure legend of the revised version of the manuscript.

4. Figure 6A: The GS staining looks quite different in Alg2 mutant as compared to WT throughout the section. This is not reflected in the description provided in the figure legend and in the authors' conclusion from this figure.
We have reworked figure 6 to allow a better comparison of wild type and mutant sections and immunohistochemistry with anti-Rx2 (cone photoreceptors, Mueller Glia cells as well as CMZ in the peripheral neuroretina) and anti GS antibodies (Mueller Glia cells) respectively. We avoided any uneven staining with the anti-GS antibody and in the new figure 6 the GS staining intensity across the entire section is fully comparable between wild type and mutant. The data presented fully support the statements made in text and figure legend in the revised version of the manuscript.

Other points
1. Title: Is humanized the appropriate term here? One usually sees this adjective when a gene or a specific cell type in an organism is replaced by the corresponding gene or cell type from humans (like a humanized monoclonal antibody or pigs with a humanized liver). I'm not sure whether this is the correct word for a knock-in allele of a gene in a model organism.
We have been following the referee's advice and have changed the title of the manuscript accordingly to "A patient-based medaka alg2 mutant…".

2. Line 45: photoreceptors "," resulting "in" retinitis pigmentosa…
edited – thanks for critical reading

3. Line 71: When glycosylation is affected in patients, "they" suffer from Congenital…
edited – thanks for critical reading

4. Line 78: "A number of" key steps … ("Few" has a negative connotation here).
edited – thanks for critical reading

5. Line 85: The preferred term for the switch in the location of LLOs is "flipping". "Internalization" would suggest something like endocytosis, but here the LLO does not lose its link to the ER membrane; only what is outside is flipped to the inside. *We thank the referee for clarification and changed the wording accordingly*

© 2021. Published by The Company of Biologists under the terms of the Creative Commons Attribution License (https://creativecommons.org/licenses/by/4.0/).

6. Line 90: to form “hybrid and” complex glycan …
added as suggested- thanks for critical reading

7. Lines 124-126: I suggest that the authors rephrase “at the basis” and the sentence after “Striking”. The messages are not clear (especially for the sentence after Striking).
We changed the respective sentences.

8. Line 130: I’m not sure why “initially” is used here.
We appreciate the referee’s comment and have rephrased the sentence.

9. Line 135-136: The first sentence of this paragraph is too broad and not accurate. There are many examples of glycosylation related proteins that are not embryonic lethal even when null alleles are used. Perhaps the authors should add “many” before “glycosylation related proteins” in this sentence.
We edited the sentence as suggested by the referee.

10. Figure 1F: Does each square/circle represent one animal? Please mention in the legend.
Yes, we added this information n = 3 (mutant); n = 4 (wt) to the legend

11. Line 197: Are homozygous mutants 100% lethal? How many animals have been used for each of three mutants generated in this study to reach this conclusion?
Indeed, homozygous mutants die 2-3 days post hatching, so the mutation is homozygous juvenile lethal. We now include the respective numbers in Table S1 presenting individually genotyped hatchlings at day 4 post hatch of two batches descending from heterozygous alg2$^{p.G336*/+}$ crosses. From a total of 80 embryos, 29 had died until 4 days post hatching. The remainder was individually genotyped resulting in 20 wildtype, 31 heterozygous and 0 homozygous mutant hatchlings. We now include this statement in the first results paragraph describing the mutant.

12. Line 237: “Fig. S2” instead of Fig. 32.
Thanks for notifying, we have corrected that in the revised version of the manuscript

13. Lines 383-283, Figure S3: How many animals have the authors analyzed to reach the conclusion that white matter is reduced?
We saw a repeated effect (n = 2) in the homozygous and show one representative picture in Figure S2. We have toned down this statement in the maintext.
Was the phenotype 100% penetrant? It’s better to mention the animal numbers for staining data throughout the manuscript.
The phenotype was 100% penetrant. We have taken care to include the respective numbers in the figure legends or main text.

14. Lines 508-510: Alternatively, the retinitis pigmentosa phenotype might be specific to fish.
Yes, we hence have softened this sentence. We have incorporated this to the revised version of the manuscript.

15. Line 522: Perhaps “as well as” should be used instead of “as much as”. *We have been following the referee’s advice and have changed the phrase accordingly.*

16. Line 524: “all human phenotypes” seems to be somewhat exaggerated. Perhaps the authors can list the specific phenotypes that are recapitulated in their model.
We changed this following the referee’s suggestion to a more specific list.

17. Lines 569-571: Listing cytoplasmic proteins like Arrestin here can be misleading. The sequon for N-glycosylation is likely to be only meaningful for proteins that go through the ER. It’s also better to end this paragraph with a concluding sentence.
We thank the reviewer for the suggestion and have changed the text accordingly.

© 2021. Published by The Company of Biologists under the terms of the Creative Commons Attribution License (https://creativecommons.org/licenses/by/4.0/).

Reviewer 2

Advance Summary and Potential Significance to Field:
This is a well-executed study which model the ALG2-CDG in fish by introducing a mutation at the same position in the alg2 gene as an human index patient.
Homozygous mutants displayed abnormal facial skeleton, cardiovascular system rod photoreceptors - and the resulting phenotype recapitulates ALG2-CDG patients.
Proteome analysis indicated compensatory expression of glycosylation enzymes. Rod cells in the retina and altered abundance of glycoproteins directly implicated with photoreceptor function was observed. This study makes a good case to use this line as a disease model for ALG2-CDG and retinitis pigmentosa. Since the effects can be effectively rescued by mRNA injection it will also serve as an efficient tool to test the activity of modified mRNA variants.

Reviewer 2 Comments for the Author: Minor points:
1.I lack a discussion on the effects on the truncated protein; what active sites are missing etc.
This is an interesting point raised by the referee. Even though highly relevant, there are no structural data available for Alg2 except for the apparent presence of a single transmembrane domain. Consequently, the only point of reference highlighting relevant and potentially dispensable domains are mutations in human patients. The allele we modelled in medaka based on the human mutation all affect the C-terminus and all result in a comparable mutant phenotype. The fact that we can rescue the mutant phenotype provides a handle for a future systematic mechanistic analysis correlating the (primary) structure and function of the protein that we discuss in the manuscript.

2. One can assume that ALG2 is ubiquitously expressed in Medaka as well as other vertebrates – but is this investigated in the fish model (eg Medaka or Zebrafish)? Or is this assumption built solely on interference from mammalian studies?
The medaka alg2 gene is ubiquitously expressed at low levels as shown by whole mount in situ hybridization by Jana Fuhrmann in the laboratory. This is well in line with the mass-spec data showing a low abundance also on the protein level.
Figure for the referee: Whole mount in situ hybridization to detect alg2 transcripts in medaka stage 30 embryos

© 2021. Published by The Company of Biologists under the terms of the Creative Commons Attribution License (https://creativecommons.org/licenses/by/4.0/).

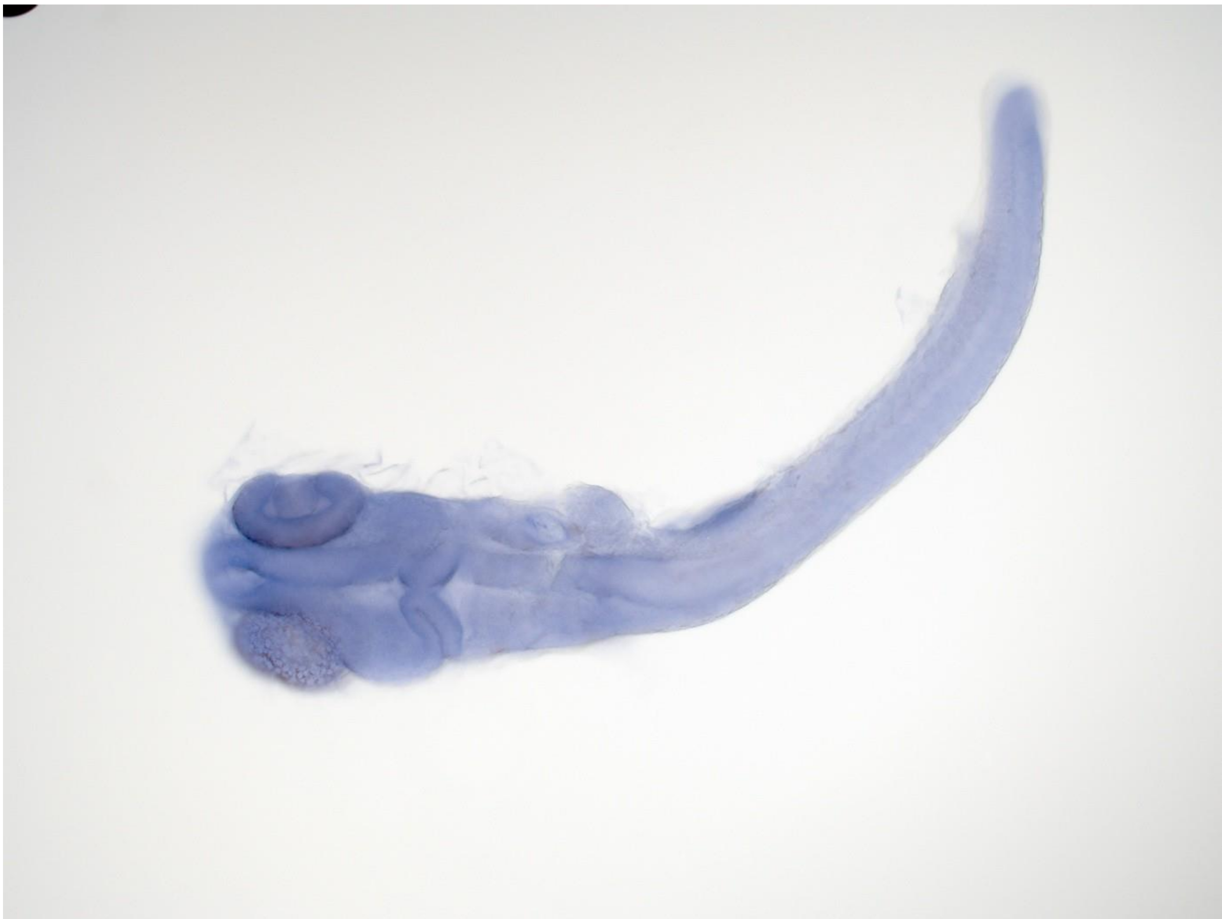

3. Mutant embryos injected with wt mRNA survive at least to day 18dph (row 475). Assuming that the phenotype is largely normal - the larva swimming and eating – does this result suggest that ALG2 might be dispensable for adult life in Medaka? Was no mRNA injected mutant ever followed to adult stages?
This is an interesting point that we are currently pursuing futher (see response to referee 3 for the rescue experiments). Rescued fish were raised but eventually died in the initial round of rescue experiments. This could be due to a requirement of ALG2 in adults as much as to a reduced level of high intensity husbandry that we were facing under Corona conditions at this time. In the absence of robust data vigourously addressing this point in long term experiments we would prefer not to discuss this (exciting) possibility in the revised manuscript.

Reviewer 3 Advance Summary and Potential Significance to Field: Manuscript by Gücüm,et al., “A humanized medaka alg2 mutant as novel model for hypo-N-glycosylation” addresses the role of ALG2 mannosyl transferase in medaka
animal model during development using CRISPR edited genetic knock in lines and a range of biochemical and developmental genetic approaches. In addition, proteomics and glycan analysis in patient fibroblasts was performed. The study is of general interest to clinicians and developmental biologists and will expand knowledge on the role of ALG2.

Reviewer 3 Comments for the Author:
The manuscript is neatly written and data are nicely presented and analyzed. However, there are numerous points that need to be address to clarify results and align claims with presented data.

Major points:
1. The model: The study presents generation of a new medaka fish model for a congenital disorder of glycosylation (CDG-Ii) by ssODN CRISPR editing to introduce a stop codon in medaka alg2 gene in an equivalent position to human ALG2 that was observed in a patient. Several alleles are induced and one is analyzed. This is very nicely done, however some clarifications throughout the

© 2021. Published by The Company of Biologists under the terms of the Creative Commons Attribution License (https://creativecommons.org/licenses/by/4.0/).

manuscript are needed. First, it is not generally accepted to refer to this type of a model as "humanized medaka model". To be accurate, the authors would need to generate a medaka model where the endogenous alg2 locus is replaced by the human ALG2 gene that carry the patient's variant. Alternatively, the authors could change the title.
We have been following the referee's advice and have chaned the title to avoid the misleading term "humanized". Instead we used "patient-based" in the title and throughout the revised version fo the manuscript.

Second, it is not clear from the results section why this (ssODN) approach was used. *We thank the referee for this point that we are happy to better explain in the revised version of the manuscript. When we started the project the ssODN approach was the best way to introduce specific modifications into the medaka genome. We used the patient's allele and designed a ssODN to introduce the patient-based mutation into the alg2 orthologue. Alongside the desired integration of the ssODN, NHEJ triggered indel formation occur as well and survival of the injected organism selects for (hypomorphic) alleles. This resulted in the 3 different alleles described in the manuscript, all mutated at the intended site. We have rephrased the respective section to better explain the reasoning.*

2. It is confusing throughout the manuscript what the authors have concluded about the nature of the model. They refer to it as viable but homozygotes die at early larval stages. It cannot be both ways.
The referee raises an important point and we have made sure to clearly describe the nature of the mutation: The mutation is recessive, early juvenile lethal and homozygous fish die 2-3 days after hatching, as we had been describing in the submitted version. To further clarify that point throughout the manuscript we now depict homozygous mutants as $alg2^{p.G336*/p.G336*}$ *to replace the misleading proxy* $alg2^{hypo}$ *for homozygous mutants used in the initially submitted version of the manuscript.*
The mutation does not cause any haploinsufficiency and heterozygous animals are viable and don't show any phenotype. This allows to keep a viable and stable heterozygous stock ($alg2^{p.G336*/+}$*) that upon incross results in 25% homozygous* $alg2^{p.G336*/p.G336*}$ *fish (Mendelian segregation). The offspring of heterozygous parents was individually genotyped and used to describe the phenotype and to run the rescue experiments respectively. We have made sure that this is clearly stated in the main text and explained in detail in the materials and methods section.*

There is also no information on the patient variant (beyond the reference) and its impact on protein presence. Is it a hypomorph or a hyploinsuficiency? Is there any evidence to substantiate it or it is unknown. My own literature search showed that this model is not faithfully recapitulating the patient situation because the patient is a trans-heterozygote for a variant leading to a stop codon and paternal variant that appears to be unstable and not contributing enzymatic activity. All these are very important nuances that could shed light on the ALG2 function and need to be addressed and discussed in the manuscript. In this context medaka model would be only testing the homozygote recessive version of one of the patient alleles.
The reviewer touches an important point. The described medaka model is not a copy of the human situation and not intended to be. We instead used the position of the ALG2 mutation in the maternal allele of the index patient as basis to establish medaka alleles that allow testing the developmental consequences of these patient- based alleles under conditions of homozygousity. In the revised version of the manuscript we have made sure to clearly state that aim.

3. The characterization of the phenotypes: The claims are overreaching, e.g. "fully resemble the multisystemic phenotype" (120) while we later learn that only a few phenotypes were tested and quite superficially described. This needs to be addressed throughout the text.
We followed the advice of the referee and have taken care to moderate and change our claims throughout the revised version of the manuscript.

Specifically, one of the core components of the CDG-Ii (and other CDGs) is myasthenic syndrome. This is not discussed or analyzed in any way. It would be extremely valuable to evaluate muscle markers and structure in this model. Do older hatchlings move in a normal way?

© 2021. Published by The Company of Biologists under the terms of the Creative Commons Attribution License (https://creativecommons.org/licenses/by/4.0/).

Unfortunatley it did not become fully apparent in the submitted version that we were unable to perform the suggested analyses since the alg2$^{p.G336*/p.G336*}$ fish die latest at day 3 after hatching. To still touch this point, we took advantage of the proteins represented in our proteomics analysis and compared them to known proteins affected in the myasthenic syndrome (linked genes by uniprot: https://www.uniprot.org/uniprot/?query=annotation%3A%28type%3Adisease+%22myasthenic+syndrome%22%29&sort=score). Our proteome analysis pinpointed the absence of Vamp1 and Slc25a1(involved in the myasthetic syndrome) from the alg2$^{p.G336*/p.G336*}$ samples. We now state that point in the revised version of the manuscript.

4. The eye phenotype is very interesting and has not been associated with ALG2 clinical symptoms, although CDGs commonly affect vision in affected persons. The authors state in the discussion that they "have shown that in alg2hypo mutants rod photoreceptors are initially born, but not maintained". This is not fully supported by the data presented. Time course imaging of retinal IHC would be needed to show the initial presence of rod photoreceptors, followed by progressive loss over time. The study would benefit from time course imaging and quantitative assessment.

The referee is right and we have taken care to conduct and incorporate the respective analyses. We have included a new figure 5, figure 7 and supplemental figure S4 to extensively address these points. We now show the time course of retinal/photoreceptor differentiation in figure 5 and supplemental figure S4 comparing the homozygous mutants to wild type siblings from the same batches. We use DAPI nuclear staining to highlight retinal lamination complemented by staining for rod photoreceptors (Rhodopsin) and cone photoreceptors (Zpr1). Rod photoreceptors are initially born and subsequently reduced and eventually lost in the central retina, while cone photorecpetors remain uaffected. Already at the onset of cone differentiation we observe an apparent failure of the mutant photoreceptor layer (comparefigure 5B/F) to segregate into an inner rod and outer cone layer. We have extensively revised the corresponding paragraph in the revised version of the manuscript to highlight the failure to eventually maintain initially born rod photoreceptors. In the new figure 7 we correlate the disappearance of rod photoreceptors to the progressive detection of apoptotic cells after the onset of the expression of rod specific markers. This supports the statement of the initial birth of rod photoreceptors that are not maintained subsequently.

5. The rescue: This is a very important part of model validation and it clearly shows that WT medaka mRNA can reverse some aspects of the overall gross morphology phenotype. The human ALG2 rescue with n=2 is substandard. Appropriate power analysis needs to be conducted and adequate number of animals analyzed. Alternatively, this can be removed along with the claims accompanying it (e.g. 525). How similar are the human and medaka proteins? Does a patient mRNA variant rescue the medaka phenotype? This would be a very important experiment especially if evaluated by biochemical or cell-level readouts.

We have introduced a direct comparision of the Alg2 proteins into Figure S1.
We agree with the referee that the rescue experiment is a very important part of the validation of the model. We have performed the rescue experiments by blindly injecting into the offspring of heterozygous parents and have subsequently raised the embryos and young fish to day four after hatching, a time point at which uninjected alg2$^{p.G336*/p.G336*}$ fish have all died. We have individually genotyped all surviving fish at this time point and while in the controls homozygous alg2$^{p.G336*/p.G336*}$ fish did not survive, the mRNA injection resulted in a rescue efficiency of about 25% at day 4 post hatching. We substantially extended the rescue analyses, both for the rescue with the mRNA encoding medaka Alg2, as well as with the corresponding human mRNA. While the medaka mRNA showed a rescue efficiency of close to 100% (Mendelian segregation of all three genotypes), the human mRNA showed a slighty reduced rescue efficiency. This is all presented in an extended figure 8 and table S1. The testing of the rescue capacity of the human mutant mRNA is an exciting experiment that we have high on our agenda for future experiments. It requires a substantially expanded colony of alg2$^{p.G336*/+}$ fish that we could not establish in the time available for the revision given the limitations we are working under in Corona.
As pointed out in the final paragraph of the discussion we consider this an interesting aspect for future analysis.

© 2021. Published by The Company of Biologists under the terms of the Creative Commons Attribution License (https://creativecommons.org/licenses/by/4.0/).

6. Glygo- and proteomics analyses: Very elegant study of glycosylation and proteomics is presented. What is the ALG2 protein level in patient fibroblasts and alg2 in medaka samples? This would be important to discuss in the main manuscript. *This is a good point. Since the Alg2 protein is a low abundance protein, it can neither in patient fibroblasts nor in medaka samples be detected by the routine GC/MS performed here (Wilhelm et al., Mass-spectrometry-based draft of the human proteome. Nature. 2014 May 29;509(7502):582-7). We discuss low level expression of the Alg2 protein and its high stablity in the context of the rescue experiments in the manuscript.*

Minor points:
1.(178) How is the SL defined? Methods section or legend would benefit from description.
We have added that to the materials and methods section: the StandardLength ist the distance between the lenses that is normalized to the total body length as described in detail in the paper cited.

2.(189) “enlarged liver” is not defined by any marker analysis or quantification.
The liver is a prominent structure at the stage of analysis. Our statement is a rather qualitative statement apparent from the images.

3.(197) Is there evidence that medaka hatchlings need blood circulation at this stage to survive?
This is an interesting question and the answer is straight-forward: blood circulation is not needed for survival at this stage as indicated by extended cardiac arrest as for example used for the imaging of the heart not impacting on the overall survival of embryos.

4.(207) Needs language editing “supposed to suffer”, Also, (33), (358), and others.
We have taken care of the language in the mentioned lines.

5.(280) The use of “hypo-N-glycosylation” is very effective, but I cannot understand why the authors use proxy for homozygote mutant. Very confusing and needs to be revised.
We follow the referee’s advice and have changed the entire text to avoid this potential confusion. We now depict the homozygous mutants as $alg2^{p.G336*/p.G336*}$*, the heterozygotes as* $alg2^{p.G336*/+}$ *and the wild type as* $alg2^{+/+}$ *whenever individually genotyped siblings are mentioned.*

6.(302) Cartilage typically is made of type II collagen and others, but type I is only marginally involved.
we thank the referee for this comment and have changed the manuscript accordingly.

7.(303) Reference typo
Corrected Shi et al., 2019

8.(328) Sec22b and Sec23a are both trafficking machinery components and are cytosolic proteins when inactive. Sec23a is clearly not processing proteins in ER. Revise here and in all other places.
We thank the reviewer for pointing out this mistake. In the text we had correctly listed Sec22b and Sec23a as part of ER-to-Golgi trafficking. We have now adjusted Figure 4 to clearly reflect that point.

9.(401) The WT is not matching the plane of section to mutant, so the two images are not comparable.
The referee is correct and we have replaced this panel with an all new immunofluorescence analysis on the time course of retinal development and lamination (new Fig. 5). The result stays the same: cone photoreceptors are massively affected in the mutant eyes (see also comments to major point 4 above)

10.(408) The layers appear to be about 30% thinner, this is more than just “slightly” thinner. Quantification of sufficient number of animals would be needed.
We have replaced the panel (see above) and removed the statement referring to the thicknes of the layers.

© 2021. Published by The Company of Biologists under the terms of the Creative Commons Attribution License (https://creativecommons.org/licenses/by/4.0/).

11.(423) What is the nature of Rx2? Some explanation would help.
We have introduced the retina specific homeobox protein 2 (Rx2) as as transcription factor expressed in stem and progenitor cells of the retina as well as in cone photoreceptors and Mueller Glia cells in the revised version of the manuscript.

12.(431) It is very hard to see anything in these images. Using gray scale in individual staining panels and color in merged would help with assessment of data. *We followed the referee's advice and have replaced the figure in question by a new Figure 7. TUNEL panels are shown in grey scale and inverted to retrieve the maximum contrast for the reader.*
In B I see a clear difference in packing of cell, tight pack in WT, diffuse but patterned retina in mutant. Number of layers are distended as seen in DAPI. What does it mean?

The referee points to an interesting aspect and we mention the potential impact of N-glycosylation on tissue integrity that becomes apparent in the retinal lamination of the outer nuclear layer in the mutant.
Conclusions should be saved for discussion and not figure legends. Here, I would rather see a brief explanation of tools or methods to help me evaluate the data.
We have fixed that.

13.(465) How much mRNA was actually injected to each embryo?
We follow the standard microinjection procedures for medaka. Approximatley 1/6 of the cell volume (500pl) are injected routinely. For reference the concentration of RNA is provided as standard in the medaka field. We present these data and the result of the rescue upon individual genotyping in figure 8 and table S1.

14.(487) Overexpression of Alg2 in WT – how was it evaluated? What were the readouts?
We injected the same amount of mRNA into all embryos originating from a cross of heterozygous parents. All embryos were injected irrespecive of their genetic constitution. To control for correct injection, GFP mRNA was used in the injection mix as injection reporter, i.e. only positively (green) embryos were selected and continued with. Once the injected hatchlings survived day four post hatching they were individually genotyped or continued to later (day 18) analysis and genotyping.
While the injection of mRNA rescued the mutant phenotype, it did not impact in any apparent way onto the development of the heterozygous or wild type embryos.

15.(592) Revised "sacrificed" (religious or cult term) to "euthanized" or "reach experimental end point"
Sacrificed is used as common term in the literature of developmental studies, while euthanized has a bad connotation in Germany and is avoided therefore. We followed the referee's suggestion changed the term to "reached experimental end point".

16. How were the lectin-blotting experiments conducted and quantified?
As for all experiments presented, the experimental procedures are described in detail in the materials and methods section.

17. Figure 3E-F: the graphs axes scale don't match up with the text. It's unclear whether there was some sort of normalization.
We have reworked the X-axis labeling and indicate log2 fold change.

18. This is very confusing: "we accordingly use alg2hypo as a proxy for the medaka homozygous mutant" Is the homozygous mutant a hypomorph? Or are the heterozygous mutants used as the hypomorph?
We have resolved this confusion (see above, point 5). To avoid a potential confusion, we now depict the homozygous mutants as $alg2^{p.G336*/p.G336*}$*, the heterozygotes as* $alg2^{p.G336*/+}$ *and the wild type as* $alg2^{+/+}$ *whenever individually genotyped siblings are mentioned.*

19. Are there any phenotypes in the heterozygous mutants?
There are no phenotypes observable in the heterozygous mutants.

© 2021. Published by The Company of Biologists under the terms of the Creative Commons Attribution License (https://creativecommons.org/licenses/by/4.0/).

20. Figure 6: It is unclear from the legend what glutamine synthase is labeling. It is difficult to see the bilayer of cells referred to in the text.
Glutamine synthetase (GS) is used as a marker to stain the Müller Glial cells in the central retina as described in the figure legend.

Second decision letter

MS ID#: DEVELOP/2020/199385

MS TITLE: A patient-based medaka alg2 mutant as novel model for hypo-N-glycosylation

AUTHORS: Sevinç Gücüm, Roman Sakson, Marcus Hoffmann, Valerian Grothe, Clara Becker, Kaisa Pakari, Lars Beedgen, Christian Thiel, Erdmann Rapp, Thomas Ruppert, Thomas Thumberger, and Joachim Wittbrodt
ARTICLE TYPE: Research Article

I am happy to tell you that your manuscript has been accepted for publication in Development, pending our standard ethics checks. The referee reports on this version are appended below - you will note that one reviewer suggests adding an "a" to the title.

Reviewer 1

Advance summary and potential significance to field

The authors have done a great job in addressing the points raised in the first round of review. They have performed a significant number of new experiments and have revised some parts of the text to incorporate the new data and to address the comments raised by me and other reviewers. I think the manuscript is significantly improved and will be read with great interest by Development's readership.

Comments for the author

The only suggestion I have for the authors is to consider adding an "a" before "novel model for hypo-N-glycosylation" in the title.

Reviewer 2

Advance summary and potential significance to field

See previous comments

Comments for the author

I find the authors response and explanations satisfactory, no further comments.

Reviewer 3

Advance summary and potential significance to field

This study adds previously unknown phenotypic information on the role of Alg2 in eye development and maintenance.

© 2021. Published by The Company of Biologists under the terms of the Creative Commons Attribution License (https://creativecommons.org/licenses/by/4.0/).

Comments for the author

In revised manuscript entitled “A patient-based medaka alg2 mutant as novel model for hypo-N-glycolylation”,
the authors have addressed our comments satisfactorily. They should be commended for their earnest efforts to improve it.
The title was modified and now it better reflects the manuscript's content. Further, newly added data on photoreceptor development and maintenance are compelling and extend the pathological mechanism of the CDG-Ii.

© 2021. Published by The Company of Biologists under the terms of the Creative Commons Attribution License (https://creativecommons.org/licenses/by/4.0/).
